# Chemical characterization of green liquor dregs from 16 Swedish pulp and paper mills between 2017 and 2019

**DOI:** 10.1007/s11356-024-34074-3

**Published:** 2024-07-03

**Authors:** Nanna Stahre, Lotta Sartz, Mattias Bäckström

**Affiliations:** 1 Man-Technology-Environment Research Centre, Örebro University, 701 82 Örebro, Sweden; 2 Bergskraft Bergslagen AB, Södra Kungsvägen 49, 692 30 Kumla, Sweden

**Keywords:** Green liquor dregs, Mine waste, Co-disposal, Paper pulp industry, Remediation, Mine site remediation mine site reclamation, Acid mine drainage, Acid rock drainage

## Abstract

Green liquor dregs (GLD) is an alkaline by-product from the pulp and paper industry with a pH between 10 and 14. Today most of the produced GLD in Sweden is landfilled. As a fine-grained alkaline material, it might be possible to use it for acid-generating mining waste remediation. To increase the utilization, quality characteristics and environmental performance need to be determined. In this study samples were collected 5 times from 16 mills during a period of 2.5 years, and were characterized by analyzing dry matter content, loss on ignition (LOI) 550 °C and LOI 950 °C, elemental analysis, pH, electrical conductivity, and calorific value. The results were then evaluated using multivariate statistics (PCA) as well as being compared to other studies and Swedish till. The results show that even if GLD is heterogenous (both within a mill and between different mills) trends can be seen for samples from most mills. When samples do stand out, it is predominately related to the same four mills. Most of the studied parameters showed characteristics favorable for use as a remediant; however, TOC, sulfur, and some of the elements require further study. In general, this study concludes that GLD can be a viable option for the remediation of small orphaned sulfidic mining sites and thus worthy of further studies on the interaction between GLD and acidic mining waste.

Overall, GLD can be a good alternative for cost-effective remediation of smaller orphaned mining sites. It is readily available in large quantities, has the qualities needed for remediation of many orphaned acidic mining sites, and can often be locally sourced near the mining site. The use of GLD for mining site remediation is likely also a more sustainable method compared to traditional remediation methods.

**Supplementary Information:**

The online version contains supplementary material available at 10.1007/s11356-024-34074-3.

## Introduction

Green liquor dregs (GLD) is an alkaline by-product from the pulp and paper industry with a pH of 10–14. It is heterogeneous (Rihm et al. 2009), mainly consisting of non-process elements (NPE) from wood pulping, organic material, and spent cooking chemicals as well as calcite from added lime mud (Pöykiö et al. 2006; Mäkelä et al. 2016). At 35% of the total waste generated (SGI 2003), it is the largest waste fraction from the pulp and paper industry in Sweden with approximately 110,000 metric tonnes dry weight produced annually (Mäkelä et al. 2016). Today most of the produced GLD in Sweden is landfilled and only a small portion is utilized. Low utilization poses a future problem, as several mills are facing full landfills in the future. Permits to open new landfills are hard to obtain with heavy regulation as well as a lack of available land. There have also been discussions in Sweden on imposing a landfill tax on GLD and landfill taxes in Europe are generally increasing (Hoogmartens et al. 2016). As GLD can contain 40–75% water, this can become an economical problem for the mills. Kinnarinen et al. (2016) recognized that sooner or later utilization of pulp and paper waste, GLD being a major fraction, will be a more viable and economical alternative than landfill disposal.

At the same time, Sweden has several thousand small orphan sulfidic mining sites posing an environmental problem. Several of these sites produce acid rock drainage (ARD), with low pH and high trace element contents (Kargbo et al. 2004; Nyström et al. 2019; Alakangas et al. 2014). Remediation of these sites is both an economical and practical problem as conventional treatment is expensive at a larger scale (Moodley et al. 2018). Since the mining sites are orphan (SGU 2014b), society or the current landowner must also bear the cost of remediation. Many sites are inaccessible and may have cultural heritage marked features, meaning that remediation may be limited to non-appearance changing techniques. The sheer quantity of old mining waste and old mining sites, as well as the placement of the waste with relatively small amounts at each site, means that waste utilization as a secondary resource as described by Park et al. (2019) is not feasible today.

Thus, Sweden has a problem with two different types of waste: acidic waste that leaches trace elements due to the low pH and alkaline waste that leaches trace elements partly due to high pH and usually has a high buffering capacity. Most trace elements have leaching minima close to neutral pH, with higher leaching at both more acidic as well as more alkaline conditions. An adjustment of pH for both wastes to near neutral will both decrease the environmental impact of debilitating pH and decrease environmental impact due to trace element leaching to the recipient. This presents a scenario where two very different kinds of waste, which are both problematic due to their opposite characteristics, can be co-disposed. The resulting combined waste has a near-neutral pH, and an effluent that has a less severe environmental impact than the separated effluents from either waste (Bellaloui et al. 1999; Jia et al. 2019). The use of GLD for mining site remediation also has the added environmental benefit of decreasing land used for landfilling (Catalan and Kumari 2005).

Prior research has found that GLD is suitable for remediation of acid mining waste and acid rock drainage by means of cover (by itself or as an additive), injection, or co-disposal (Bäckström et al. 2010; Chtaini et al. 2001; Jia et al. 2013; Jia et al. 2014; Jia et al. 2017; Jia et al. 2019; Mäkitalo et al. 2014; Mäkitalo et al. 2015; Mäkitalo et al. 2016; Ragnvaldsson et al. 2014; Sartz 2010; Sartz et al. 2010; Sartz et al. 2018). This is due to its low hydraulic conductivity, high pH, high buffering capacity, and ability to stick to the mining waste it is injected into and not being washed out. Injection does not alter the visual appearance of the site and can be done with a smaller rig, meaning better accessibility to small sites (Sartz 2010; Sartz et al. 2018).

However, all prior studies on GLD typically only involve a few samples from one or two different mills, and data for most elements are often lacking. For example, there is a shortage of data on carbon species in GLD, calorific value, and variation in element concentrations over time and between mills. Because of this lack of data, only a few generalized characteristics are specified for GLD. In Sweden, this is a problem from a remediation perspective as there usually are two legislative options for remediation: (1) the use of a well-recognized technique or (2) the use of a more novel technique. For a remediation technique to be considered recognized, a large-scale basic characterization of several representative samples, taken during a longer period to determine large-scale variability in the population, is necessary. Data published on GLD so far is not enough to determine this variability. This study will provide a large enough sample population to take the first steps towards making use of GLD as a well-recognized technique.

## Generation of green liquor dregs in the pulp and paper mills

Green liquor dreg (GLD) is a waste product generated by both the sulfate and sulfite pulping processes in the pulp and paper industry. In the sulfate pulping process (also called Kraft process), the cooking chemical, called white liquor, consists of NaOH and Na_2_S, and pH is alkaline, in general at or above pH 10. In the sulfite pulping process, the cooking chemical consists of Mg(HSO_3_)_2_ and pH is low, around pH 4. GLD mainly consists of the non-process elements (NPE) and wood constituents that remain after the pulp has been separated out from the cooked wood. It is most often black, but color variation such as greenish gray or even bluish gray occurs.

**Fig. 1:**
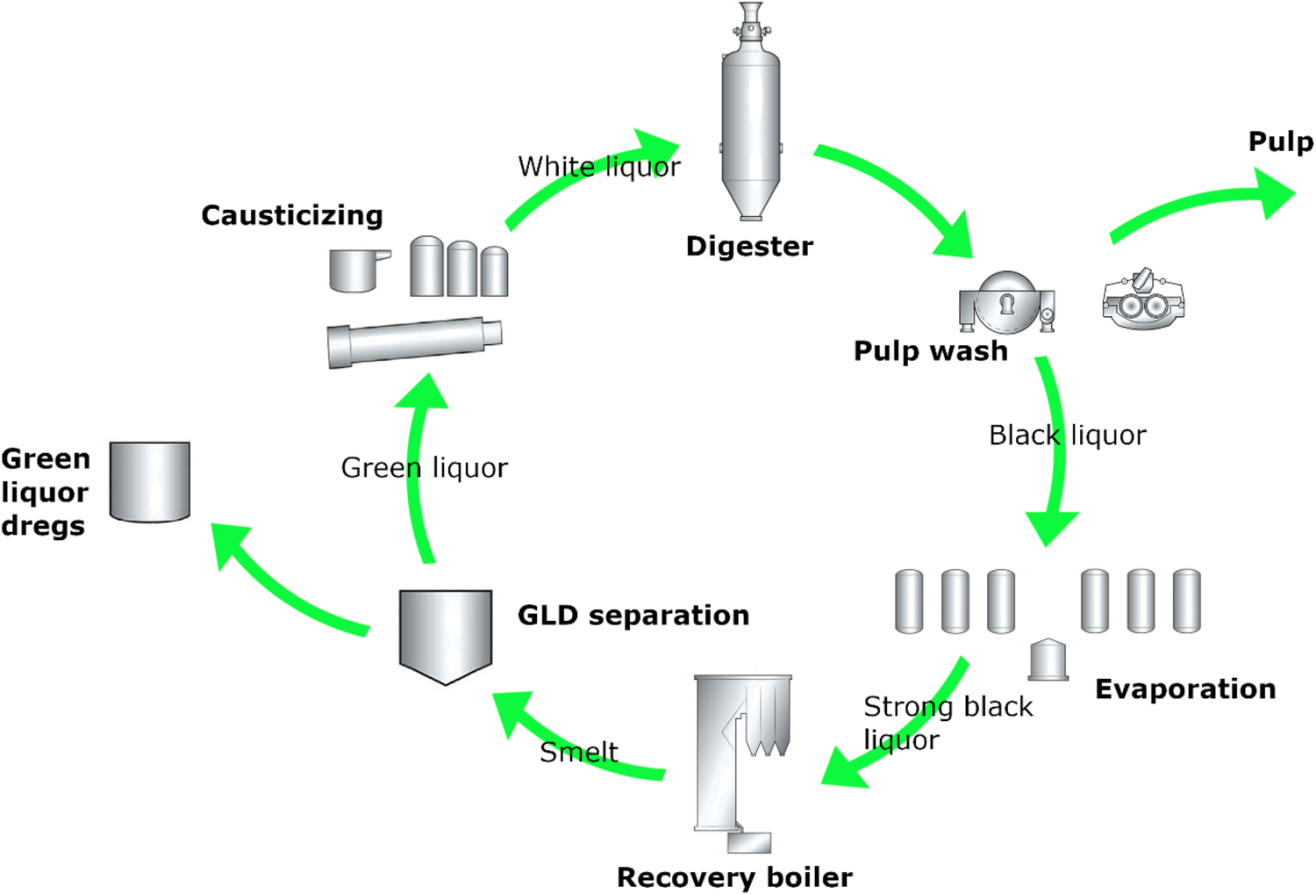
Schematic of the liquor cycle in a sulfate mill. Digestion and pulp wash considered cooking, evaporation, recovery boiling, and GLD separation, which are the main steps of the chemical recovery path where GLD are separated out from the system, and finally, there is causticizing before cooking starts again

The pulping process is generally divided into four steps: (1) wood processing, (2) cooking, (3) product-specific processes, and (4) chemical recovery. Wood processing and product-specific processes are not described in this study as they have no impact on GLD. Cooking, chemical recovery, and wood species are the three main factors that affect the properties of GLD. However, from a chemical (i.e., the liquor) perspective, chemical recovery and causticizing is a more or less one closed continuous cycle where cooking (including pulp wash) and causticizing in themselves can be seen as starting and finishing steps of the chemical recovery process as illustrated in Fig. 1.

### Cooking

Cooking is either performed in batch cookers or in continuous cookers. The wood is cooked with an alkali solution called white liquor. White liquor at sulfate cooking mills is a mixture of sodium hydroxide (NaOH) and sodium sulfate (Na_2_SO_4_) (Stenmarck and Sundqvist 2006). In sulfite cooking mills the white liquor consists of either sodium sulfite (Na_2_SO_3_), magnesium bi-sulfite (Mg(HSO_3_)_2_), or calcium sulfite (CaSO_3_). After completed cooking, the pulp is separated, washed, and proceeds to product-specific preparations such as bleaching and drying. The remaining solution is called weak black liquor and consists of reacted white liquor and the remaining wood after the fibers are removed. The weak black liquor is sent to the recovery process for chemical recovery (Golmaei 2018; Sanchez 1997).

### Chemical recovery

In the sulfate and the sodium sulfite recovery process, the weak black liquor is evaporated into strong black liquor and then combusted at temperatures up to 1200 °C in a recovery boiler (Manskinen et al. 2011).

During combustion, the black liquor forms a smelt that is then dissolved with weak liquor (diluted white liquor). The resulting liquid is called green liquor due to its greenish color that comes from dissolved sodium hydrosulfide (Golmaei et al. 2018). Suspended particles in the green liquor are separated out by means of clarification, sedimentation, or filtration, and the sediment/filtrate is called green liquor dregs. The filtrated green liquor is then sent for caustization (Golmaei 2018).

After the separation of green liquor and dregs, the dregs often still contain some green liquor. To recover this remaining green liquor some mills wash the dregs with water. Dry matter content is increased by dewatering either by a lime mud precoat filter (either rotating drum or PDF filter), filter press, or centrifugation (Golmaei 2018). Two mills in the study, however, do not dewater the GLD prior to disposal.

### Causticizing

The main purpose of causticizing is to convert Na_2_CO_3_ back into NaOH to be used again as a white liquor cooking chemical (Mäkela et al. 2010). Clarified green liquor is mixed with lime to produce white liquor (NaOH) that is then separated out in a clarifier and reused as a cooking chemical. The residual is called lime mud (CaCO_3_) and is either used in different filters during GLD processing or reburned into lime in a lime kiln (Golmaei 2018; Sanchez 1997).

## Method

### Sample collection

**Fig. 2:**
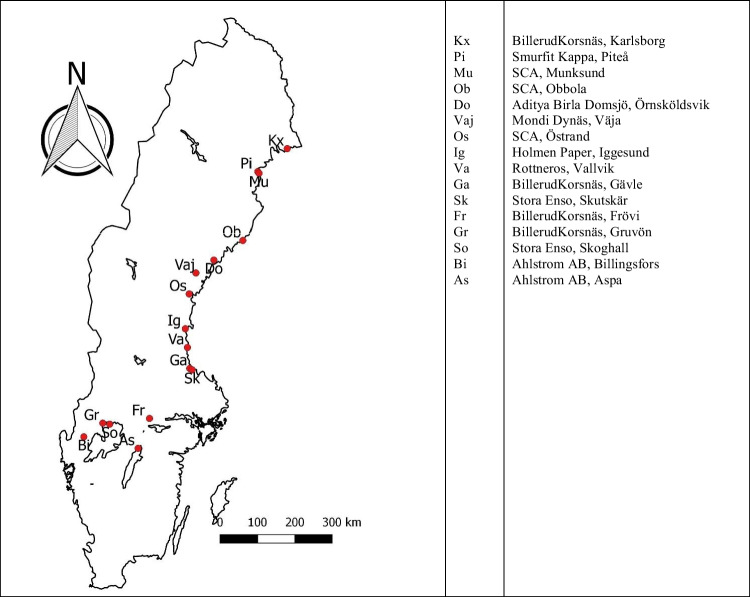
Map over locations of the participating mills in Sweden

Samples were collected twice in 2017 (spring and fall), twice in 2018 (spring and fall), and once in 2019 (spring). Sampling was performed by the mills themselves at the end of the GLD processing line. For the location of the mills, see Fig. 2. Each sample had, on average, a volume of 4–6 L. In total 71 samples were analyzed (Table 1).

**Table 1:** Received samples and sample type

Mill	Sample series				
	Spring 2017	Fall 2017	Spring 2018	Fall 2018	Spring 2019
BillerudKorsnäs Karlsborg	Single	Single	Single	Single	Single
Smurfit Kappa Piteå	No sample	Single	Single	Single	Single
SCA Munksund	Single	Single	Single	Single	Single
SCA Obbola	Single	Single	Single	Single	Single
Aditya Birla Domsjö	No sample	2 singles	Single	Single	Single
Mondi Dynäs Väja	Single	No sample	No sample	Single	No sample
SCA Östrand	Composite	Composite	Composite	Composite	Composite
Holmen Paper Iggesund	Single	Single	Single	Single	Single
Rottneros Vallvik	Single	Single	Single	Single	Single
BillerudKorsnäs Gävle	Single	Single	Single	Single	Single
Stora Enso Skutskär	Single	No sample	No sample	No sample	No sample
BillerudKorsnäs Frövi	Single	Single	Single	Single	Single
BillerudKorsnäs Gruvön	Single	Single	Single	Single	Single
Munksjö Paper Billingsfors	Composite	Single	Single	Single	Single
Munksjö Paper Aspa	Single	Single	Single	Single	Single
Stora Enso Skoghall	No sample	Single	Single	Single	Single

Most samples were grab samples while composite samples consisted of three subsamples taken during a 24-h period

Coniferous trees (pine and spruce) are the most used wood in the participating mills. Pine is generally European red pine (*Pinus sylvestris*), and spruce is generally Norway spruce (*Picea abies*); however, other species, for example, *Pinus contorta*, occur. Domsjö (Do) is a biorefinery using a sodium sulfite cooking process based on NaOH and SO_2_, creating predominately sodium bisulfite (Na(HSO_3_)_2_), but does generate unwanted thiosulfate S_2_O_3_^2−^, in the process. Gruvön (Gr) combines both the sulfite and the sulfate cooking processes: two sulfate cookers and one sodium sulfite cooker. The remaining mills only use sulfate cooking. Table 2 presents some factors that might affect the physical and chemical properties of the green liquor dregs.

**Table 2:** Process factors that might affect the physical and chemical composition of the green liquor dregs

Mill	Cooking process	Cooker type	Wood used	Green liquor separation	Washing of dregs	Dreg drying type	Produced GLD as a function of the amount of wood processed GLD*/m^3^fub m^3^fub/year
BillerudKorsnäs Karlsborg	Sulfate	Batch	70% pine, 30% spruce	X-filter	Water	Lime mud filter (precoat)	2.11 1,600,000
Smurfit Kappa Piteå	Sulfate	Continuous (3 cookers)	30% deciduous, 39% pine, 17% spruce, 14% coniferous chips	Clarifier	Water	Lime mud filter (precoat)	2.28 2,100,000
SCA Munksund	Sulfate	Continuous	40% deciduous, 60% pine	Clarifier	Water	Filterpress	0.44 1,113,000
SCA Obbola	Sulfate	Continuous	60% pine, 40% spruce	Clarifier and X-filter	Water	Centrifuge	0.51 1,030,000
Aditya Birla Domsjö	Sulfite (Na-sulfite)	Batch	At least 60% spruce, the rest pine	Sockfilter	Water	Centrifuge	1.21 1,300,000
Mondi Dynäs Väja	Sulfate	Batch	100% coniferous (pine and spruce)	Clarifier	Water	Lime mud filter (precoat)	1.07 1,100,000
SCA Östrand	Sulfate	Continuous	70 ± 10% pine, 30 ± 10% spruce	LimeGreenFilter	Water	Centrifuge	1.26 2,125,000
Holmen Paper Iggesund	Sulfate	Continuous	48% deciduous, 42% pine, 10% spruce	Clarifier	Water	Lime mud filter (precoat)	2.27 1,650,000
Rottneros Vallvik	Sulfate	Continuous	100% coniferous (pine and spruce)	Clarifier	Water	Lime mud filter (precoat)	8.41 1,093,000
BillerudKorsnäs Gävle	Sulfate	Continuous	25% deciduous, 70% pine, 5% spruce	Clarifier	Water	Lime mud filter (precoat)	5.87 2,800,000
Stora Enso Skutskär	Sulfate	Continuous	25% deciduous, 65% pine, 10% spruce	Clarifier	Water	Lime mud filter (precoat)	Not available Not available
BillerudKorsnäs Frövi	Sulfate	1 Continuous and 1 batch	37% deciduous, at least 9% spruce, 54% pine	Clarifier	Water	PDG filter with lime mud	3.03 1,150,000
BillerudKorsnäs Gruvön	Sulfate and sulfite (Na-sulfite)	3 Continuous	33% deciduous, 40% pine, 23% spruce	Cassette filter and PDG filter with lime mud	No	No dewatering	4.37 2,200,000
Munksjö Paper Billingsfors	Sulfate	Batch	20% deciduous, 80% coniferous	Clarifier	No	No dewatering	1.32 300,000
Munksjö Paper Aspa	Sulfate	Continuous	50% pine, 50% spruce	Cassette filter	Water	Larox filter	1.05 850,000
Stora Enso Skoghall	Sulfate	Continuous	60% pine, 40% spruce	Filtration (80%) and clarifier (20%)	Water	Lime mud filter (precoat)	Not available Not available

Pine is generally *Pinus sylvestris* and spruce is generally *Picea abies*; m^3^fub is a Swedish wood measurement used by the forestry and paper and pulp industry that measures m^3^ wood after debarking. The last column specifies how much GLD (in kg d.w.) each mill produces for each m^3^fub, as well as how much wood in m^3^fub each mill processes per year

Mills that have newer equipment and more efficient dewatering techniques (like the Obbola and Munksund mills) that do not require lime mud generally generate much less GLD per quantity of wood processed. The efficiency of the recovery boiler also impacts the amount of GLD produced/amount of wood processed but is not the only factor as illustrated by Obbola. They have the highest average TOC, which indicates a low recovery boiler efficiency but are still one of the mills with the lowest amount of produced GLD/amount of wood processed.

### Analysis

#### pH and electrical conductivity

To measure pH and electrical conductivity, wet samples corresponding to 5 g dry matter were placed in a 50 mL Sarstedt tube with 50 mL of MQ water and shaken in an end-over-end shaker for 2 h. After shaking, the samples were left to sediment for 20 min before pH was measured with a Metrohm 744 pH meter and electrical conductivity with a Hach sensION + EC7 meter. Calibration was performed daily using a pH 7 and a pH 12.45 buffer. The pH 12.45 buffer consisted of a saturated Ca(OH)_2_(s) solution.

#### Elemental analysis

Elemental analysis was performed on dried (105 °C) and homogenized samples. Samples were analyzed by MS Analytical in Vancouver, Canada. Total carbon (TC) and total sulfur (TS) were analyzed by a Leco carbon/sulfur analyzer. Iron and tungsten accelerators were added to the sample as a stream of oxygen passed over it in an induction furnace. Released carbon dioxide/sulfur dioxide was measured by an IR detection system and the total carbon/sulfur content was determined. Graphite was measured by first ashing the sample at 550 °C to remove organic carbon and then leaching it with dilute hydrochloric acid to remove soluble inorganic carbon. The residue was dried at low temperature and then analyzed for graphite carbon content by carbon/sulfur analyzer. Total inorganic carbon content (TIC) was calculated by subtracting analyzed graphite content from the ashed carbon content and total organic carbon content (TOC). TOC was calculated by subtracting ashed carbon content from total carbon content. Sulfate was measured by digesting a sample with dilute hydrochloric acid on a preheated hot plate. After digestion, the sample solution was analyzed by ICP-AES. Sulfide concentrations were determined by subtracting sulfate sulfur from total sulfur. For major elements samples were fused in a muffle furnace at 1000 °C with lithium borate, dissolved in dilute nitric acid after cooling, and the resulting solution was then analyzed with an ICP-OES (Thermo Scientific/iCAP 6000). Prior to analysis of trace elements and REE (Ba, Ce, Cr, Cs, Dy, Er, Eu, Ga, Gd, Hd, Ho, La, Lu, Nb, Nd, Pr, Rb, Sm, Sn, Sr, Ta, Tb, Th, Tm, U, V, W, Y, Yb and Zr), the samples were fused with lithium borate in the same way as the major elements but the smelt was diluted with mineral acids and analyzed with ICP-MS. More volatile trace elements (As, Au, Bi, Hg, Sb, Se, Tl, Ag, Cd, Cu, Mo, Ni, Pb, and Zn) were analyzed after aqua regia digestion using ICP-MS.

#### Calorific value

To independently verify the results from LOI and TOC analysis, the calorific value was also determined for samples collected during the fall of 2018. Samples were analyzed by Eurofins Biofuel & Energy Testing Sweden AB using standard protocol SS-EN 15400:2011.

#### Principal component analysis

Principal component analysis (PCA) was performed on scaled data using R with the function prcomp from the library factoextra. First all elements/features that contained values below detection limit were removed. The remaining data was then scaled by log_2_ and standardized (*z*-score standardization). Factor scores were calculated by using Thurstone’s regression-based weights (Thurstone 1947).

## Results and discussion

### Dry matter content

Dry matter varies between 25 and 75% for the different mills (Table 3). Generally, there is a low variation within a mill’s samples (< 10% standard deviation). However, two mills, Karlsborg (Kx) and Piteå (Pi), have > 10% standard deviation attributed to one sample from each mill having a significantly higher or lower dry matter content.

**Table 3:** Measured dry matter content of samples presented as average ± standard deviation (based on three replicates)

Dry matter content (weight-%)						
	Spring 17	Fall 17	Spring 18	Fall 18	Spring 19	Average
Aspa	31.9 ± 0.3	29.8 ± 0.5	32.0 ± 0.7	32.8 ± 0.7	35.8 ± 0.3	32.5 ± 2.0
Billingsfors	35.2 ± 0.6	32.2 ± 0.3	28.1 ± 0.1	36.3 ± 0.2	29.2 ± 0.1	32.2 ± 3.2
Domsjö	No sample	49.0 ± 0.5	60.0 ± 2.4	52.9 ± 0.4	55.6 ± 2.6	54.4 ± 4.0
Frövi	47.0 ± 0.3	34.4 ± 0.2	37.2 ± 0.3	40.7 ± 0.5	36.3 ± 0.2	39.1 ± 4.4
Gruvön	56.3 ± 0.4	64.3 ± 0.9	68.8 ± 1.0	67.2 ± 0.6	63.9 ± 0.2	64.1 ± 4.3
Gävle	55.8 ± 1.9	53.3 ± 0.2	59.2 ± 0.2	61.8 ± 0.7	46.9 ± 1.1	55.4 ± 5.1
Iggesund	56.3 ± 0.3	53.7 ± 1.3	57.0 ± 0.7	53.2 ± 0.6	55.2 ± 0.8	55.1 ± 1.5
Karlsborg	33.5 ± 0.4	31.7 ± 0.1	32.0 ± 0.5	59.9 ± 0.1	30.1 ± 0.6	37.4 ± 11.3
Munksund	41.6 ± 0.1	36.9 ± 2.3	47.5 ± 1.1	31.8 ± 0.1	35.8 ± 1.5	38.7 ± 5.4
Obbola	38.2 ± 0.1	36.4 ± 0.4	38.7 ± 0.0	33.1 ± 0.2	26.8 ± 0.1	34.6 ± 4.4
Piteå	No sample	50.6 ± 0.1	47.2 ± 0.2	63.9 ± 0.3	29.7 ± 0.8	47.9 ± 12.2
Skoghall	No sample	29.1 ± 0.5	45.8 ± 0.2	47.3 ± 0.3	28.5 ± 0.5	37.7 ± 8.9
Skutskär	64.7 ± 0.0	No sample	No sample	No sample	No sample	64.7
Vallvik	74.8 ± 0.0	63.0 ± 0.4	71.4 ± 0.0	72.1 ± 0.3	64.5 ± 0.1	69.2 ± 4.6
Väja	48.0 ± 13.2	No sample	No sample	30.5 ± 0.3	No sample	39.3 ± 8.8
Östrand	33.2 ± 0.1	34.0 ± 0.3	53.3 ± 0.4	35.1 ± 0.2	33.7 ± 0.4	37.9 ± 7.7

The value for Fall 17 for Domsjö is an average of both samples (based on three replicates each)

According to the mills, dry matter content is mainly a function of the state of the dewatering equipment, where age and operational status have more impact than the type of equipment. They also report that small differences in the cooking processes, such as the type of fuel used, sometimes also influence the dewatering efficiency and thus dry matter content.

### Organic matter

Despite large differences between LOI_550_ (Table 4) and LOI_950_ (Table 5) for the different samples, the total weight loss during loss on ignition for most samples is around 50%. LOI_550_ is roughly interpreted as a result of organic matter oxidation while the loss between 550 and 950 °C (LOI_950_) is roughly interpreted as a result of carbonate decomposing.

**Table 4:** Loss on ignition (LOI_550_) at 550 °C presented as average ± standard deviation (based on three replicates)

LOI 550 °C (weight-%)						
Mill	Spring 17	Fall 17	Spring 18	Fall 18	Spring 19	Average
Aspa	37.7 ± 4.8	44.9 ± 2.1	37.9 ± 3.7	39.4 ± 0.5	41.8 ± 0.1	40.3 ± 2.2
Billingsfors	24.0 ± 0.3	31.4 ± 0.6	25.2 ± 0.3	27.9 ± 0.5	29.8 ± 0.5	27.6 ± 0.4
Domsjö	No sample	8.7 ± 0.8	14.8 ± 9.5	6.0 ± 0.7	9.0 ± 4.5	9.6 ± 3.9
Frövi	12.3 ± 1.0	28.6 ± 0.4	26.5 ± 0.3	18.1 ± 0.8	23.9 ± 0.3	21.9 ± 0.6
Gruvön	6.5 ± 0.2	7.1 ± 0.6	6.0 ± 0.3	6.8 ± 0.4	6.8 ± 0.6	6.6 ± 0.4
Gävle	13.0 ± 1.4	9.1 ± 0.3	6.7 ± 0.0	6.4 ± 0.1	14.8 ± 0.3	10.0 ± 0.4
Iggesund	7.8 ± 0.3	6.5 ± 0.3	5.5 ± 0.3	8.4 ± 0.1	4.8 ± 0.2	6.6 ± 0.2
Karlsborg	23.3 ± 0.4	25.4 ± 0.8	16.7 ± 3.2	3.9 ± 0.1	26.5 ± 0.8	19.2 ± 1.1
Munksund	23.5 ± 0.4	24.8 ± 1.1	13.9 ± 0.3	15.0 ± 4.2	18.2 ± 1.5	19.1 ± 1.5
Obbola	27.4 ± 0.7	26.4 ± 0.2	27.3 ± 0.4	35.3 ± 0.4	44.9 ± 0.4	32.3 ± 0.4
Piteå	No sample	12.9 ± 0.2	13.6 ± 0.2	3.1 ± 0.8	27.4 ± 1.9	14.3 ± 0.8
Skoghall	No sample	27.3 ± 0.8	9.7 ± 0.7	6.1 ± 0.1	26.0 ± 0.8	17.3 ± 0.6
Skutskär	5.0 ± 0.2	No sample	No sample	No sample	No sample	5.0
Vallvik	4.1 ± 0.2	4.6 ± 0.3	6.0 ± 0.1	5.2 ± 0.2	4.6 ± 0.3	4.9 ± 0.2
Väja	8.6 ± 1.0	No sample	No sample	18.2 ± 0.3	No sample	13.4 ± 0.7
Östrand	17.1 ± 0.1	21.3 ± 0.9	14.5 ± 0.5	18.8 ± 0.3	18.1 ± 0.6	18.0 ± 0.5

The value for Fall 17 for Domsjö is an average of both samples (based on three replicates each)

**Table 5:** Loss on ignition (LOI_950_) between 550 and 950 °C presented as average ± standard deviation (based on three replicates)

LOI 550–950 °C (weight-%)						
Mill	Spring 17	Fall 17	Spring 18	Fall 18	Spring 19	Average
Aspa	26.6 ± 2.8	21.5 ± 3.7	25.0 ± 4.8	19.3 ± 0.2	27.9 ± 1.5	24.0 ± 2.6
Billingsfors	20.4 ± 0.3	13.4 ± 0.7	14.8 ± 2.0	16.8 ± 0.8	15.2 ± 2.4	16.1 ± 1.2
Domsjö	No sample	18.4 ± 0.3	23.0 ± 0.7	17.6 ± 1.7	18.6 ± 1.4	19.4 ± 1.0
Frövi	34.8 ± 0.6	31.2 ± 2.6	30.4 ± 0.3	31.1 ± 0.5	30.2 ± 0.2	31.6 ± 0.8
Gruvön	33.3 ± 0.2	29.0 ± 0.5	31.5 ± 0.8	32.9 ± 0.8	29.3 ± 0.2	31.2 ± 0.5
Gävle	37.4 ± 2.2	39.0 ± 0.1	39.8 ± 0.1	40.2 ± 0.0	36.4 ± 0.3	38.5 ± 0.5
Iggesund	34.2 ± 0.1	33.6 ± 0.4	34.7 ± 0.1	32.5 ± 0.1	34.9 ± 0.2	34.0 ± 0.2
Karlsborg	24.8 ± 0.1	23.2 ± 0.8	16.3 ± 1.7	38.2 ± 0.1	17.1 ± 0.7	23.9 ± 0.7
Munksund	18.7 ± 0.2	22.9 ± 1.2	19.1 ± 0.4	10.2 ± 3.4	18.4 ± 1.8	17.8 ± 1.4
Obbola	19.2 ± 0.6	21.3 ± 0.1	27.5 ± 0.2	28.9 ± 0.3	17.7 ± 1.2	22.9 ± 0.5
Piteå	No sample	34.0 ± 0.2	31.2 ± 0.2	37.8 ± 0.5	32.2 ± 0.7	33.8 ± 0.4
Skoghall	No sample	18.7 ± 1.5	30.0 ± 0.5	33.2 ± 0.1	23.5 ± 0.6	26.4 ± 0.7
Skutskär	38.5 ± 0.1	No sample	No sample	No sample	No sample	38.5
Vallvik	41.6 ± 0.0	38.3 ± 0.1	40.8 ± 0.1	41.5 ± 0.2	40.1 ± 0.3	40.5 ± 0.1
Väja	27.5 ± 1.3	No sample	No sample	20.3 ± 0.6	No sample	23.9 ± 1.0
Östrand	13.4 ± 0.9	10.8 ± 0.6	18.9 ± 0.5	8.8 ± 0.7	16.1 ± 1.0	13.6 ± 0.8

The value for Fall 17 for Domsjö is an average of both samples (based on three replicates each)

Generally, a trend can be seen where samples containing lime mud have lower LOI_550_, but higher LOI_950_, making the total LOI approximately the same for both groups. Lime mud-containing samples have higher TIC and calcium concentrations than non-lime mud-containing samples (as is expected as lime mud contains calcite). LOI_950_ is moderately correlated to TIC when calculating Pearson correlation explaining why lime mud-containing samples have higher LOI_950_. At 550 °C only CO_2_ from organic carbon is released but at 950 °C both the organic and carbonate CO_2_ are released, making up for the difference in speciation.

Non-lime mud-containing samples have a much higher fraction of organic carbon explaining why these samples have higher LOI_550_ compared to lime mud-containing samples. It is likely that the reason for lime mud-containing samples having lower TOC is the addition of lime mud after combustion. As TIC increases due to the addition of lime mud, TOC decreases as the addition of Ca and TIC acts as a dilutant, thus making TC approximately the same in both types of samples.

**Fig. 3:**
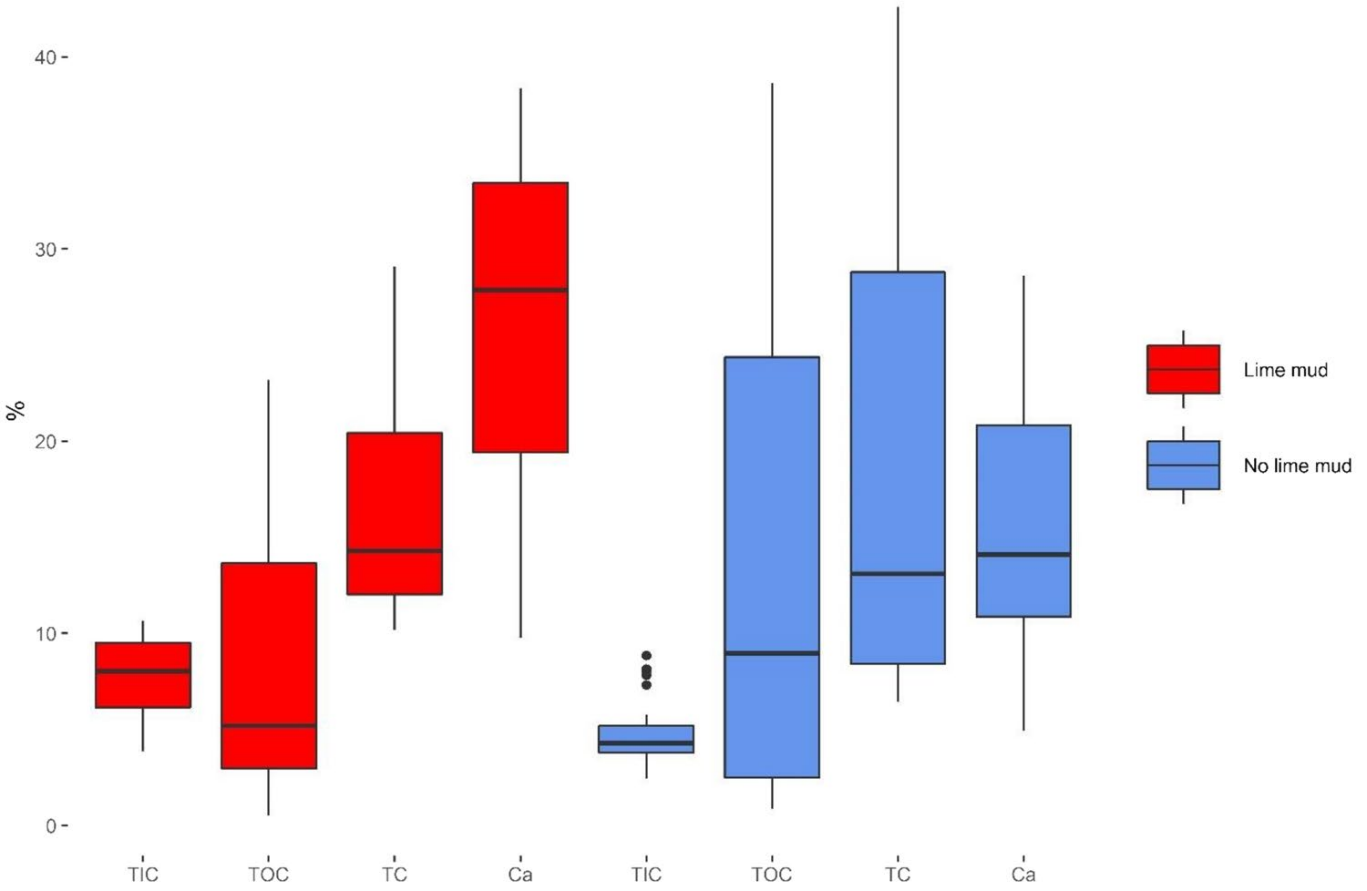
Analyzed values of the carbon species and calcium grouped by the lime mud content in the GLD. The box represents the inter-quartile range (IQR) with the line in the box representing the median, the whiskers represent either maximum or minimum when no outliers are present or the maximum/minimum value in the range of 1.5 × IQR, and outliers are represented as dots outside the whiskers. Thirty-six samples were characterized as having lime mud and 35 samples were characterized as having no lime mud

The principle of cooking chemical recovery is that all organic content is to be fully oxidized when black liquor is combusted into green liquor. High TOC and calorific value in GLD, however, indicate that this process is not very effective in some mills. Finding some remaining organic content in GLD was expected as Ribeiro dos Santos et al. (2019) also reported GLD as having organic content, believed to be in the form of lignin, cellulose, and coal. However, the high TOC concentrations in GLD from some mills are noteworthy as the concentrations are higher (in the case of Aspa much higher) than what the mills (i.e., the technical personnel) themselves believed prior to the study (Table 4 and Fig. 3).

A few samples have high calorific values matching or exceeding the calorific value of primary sludge, fiber sludge, chopped pulpwood, and pine bark confirming the presence of high organic matter content in GLD (Table 6). Primary sludge is sludge obtained from the pulp and is very similar to the pulp in composition; fiber sludge is a mixture of primary sludge, secondary sludge, and recycled paper sludge.

**Table 6:** Measured calorific values of the GLD (sampled in fall 2018) and selected reference values (Swedish Energy Agency 2017)

Mill/reference	Dried sample (*MWh/ton*)
Aspa	3.179
Billingsfors	2.378
Domsjö	0.434
Frövi	1.242
Gruvön	0.453
Gävle	0.574
Iggesund	0.525
Karlsborg	0.371
Munksund	0.777
Obbola	3.038
Piteå	0.217
Skutskär	0.259
Skoghall	0.465
Vallvik	1.134
Väja	0.779
Chopped pulpwood	2.3
Pinebark	1.55
Lignine	6
Fiber sludge	1.94
Primary sludge	2.678^a^

^a^Chiou et al. (2013)

Samples from Aspa, Billingsfors, and Obbola have the highest calorific value, and these are also the mills with the highest TOC content. TOC and calorific value have a linear correlation (*R*^2^ = 0.99) when plotted against each other (see Fig. 1 in supplementary data). This indicates that despite incineration in the recovery boiler there is a lot of organic matter left in the GLD.

The efficiency of the incineration is not only dependent on age and type of equipment as this does not explain the similarity between Obbola and Aspa. Obbola, for example, has one of the newest recovery lines of the mills participating in the study whereas Aspa has one of the oldest recovery lines in the study. In the case of Aspa, operators from the mill noticed that when fuel oil is used to support the incineration (used when the mill’s produced bark sludge cannot reach the temperature needed), it is harder to dewater the GLD after weak liquor washing. This indicates that the incineration process is sensitive to changes (as the temperature is supposed to be the same no matter the fuel type) and that these changes affect the properties of the GLD. Most likely, this is a result of fine-tuning the residence time and the temperature of the black liquor incineration. The connection between low TOC and the addition of lime mud is most likely a coincidence, but further studies are needed to confirm this. TIC and calcium content are very dependent on the addition of lime mud during dewatering but are nonetheless still present in samples where lime mud is not added during dewatering.

When TOC is > 20%, LOI_550_ is almost solely related to the TOC content. However, when TOC content is < 20%, LOI_550_ is related to more factors than TOC. PCA (see Fig. 9 in a later section titled PCA analysis) suggests that loss of sulfur species through combustion has a significant effect on LOI_550_.

**Fig. 4:**
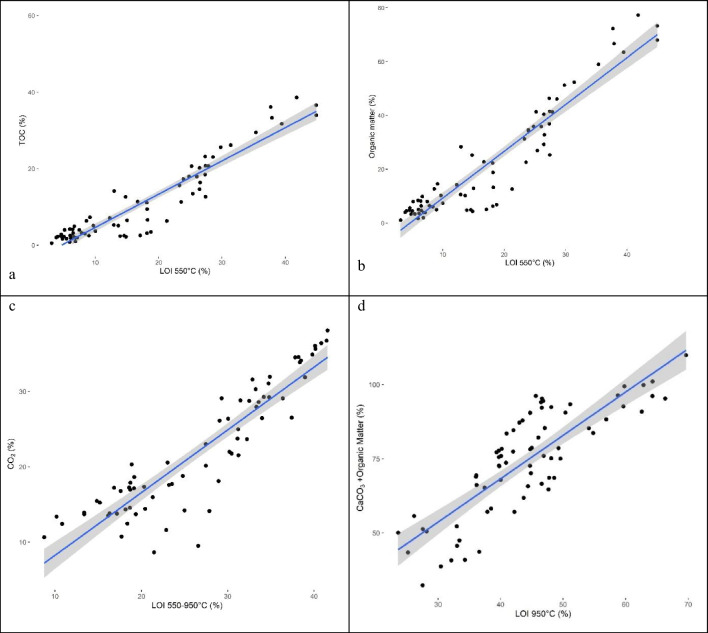
**a** LOI_550_ vs TOC (%), *R*^2^ = 0.83; **b** LOI_550_ vs organic matter (calculated as TOC × 1.724), *R*^2^ = 0.83; **c** LOI_550–950_ vs lost CO_2_ (calculated as being released from carbonates (based on TIC measurements)), *R*^2^ = 0.97; and **d** LOI_950_ vs organic matter and lost CO_2_ (calculated as being released from carbonates (based on TIC measurements)), *R*^2^ = 0.96

When comparing LOI_550_ and TOC (Fig. 4a) and LOI_550_ and organic matter (Fig. 4b) it becomes clear that the organic matter in GLD is more carbon-rich as the difference between LOI_550_ and TOC is low (Fig. 4a) and the difference between LOI_550_ and organic matter is quite large. Ordinary soil organic matter usually contains a lower fraction of carbon compared to the organic matter in GLD. In soils organic matter can usually be assumed to be approximately 70% higher than measured TOC (Heaton et al. 2016) due to the presence of oxygen, nitrogen, etc., in addition to the carbon in the large complex organic molecules (fulvic acid, etc.). In GLD there is only an excess of around 20–25% in addition to the organic carbon, indicating that the organic molecules in GLD are not as complex as the molecules found in soils. Considering that the organic material in GLD mainly consists of lignin and shorter fibers (that are excluded in pulp) and undergoes incineration in a recovery boiler as well as a further breakdown by alkali solutes in the smelting recovery, it is reasonable that even the very complex lignin molecules are degraded into smaller molecules.

**Fig. 5:**
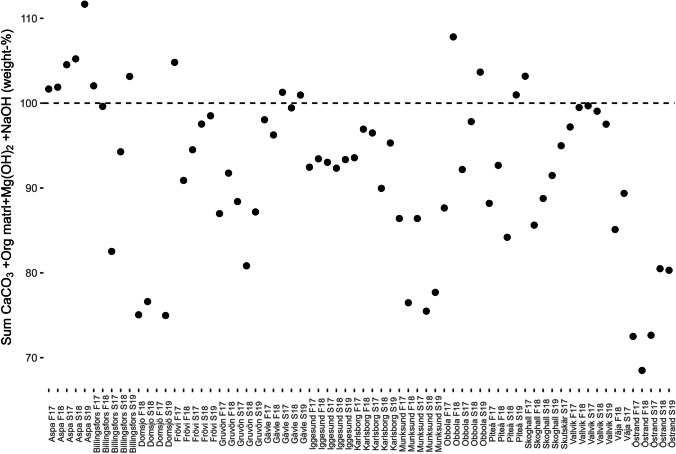
CaCO_3_ + organic material + NaOH + Mg(OH)_2_ sums up to almost the entire mass in most samples. The value for Fall 17 for Domsjö is average of both samples. Some samples sum up to above 100% due to analytical error

Comparing LOI_550–950_ and lost CO_2_ (Fig. 4c) indicates that a significant portion of the lost CO_2_ during the interval between 550 and 950 °C is being released from carbonates in the GLD. In Fig. 4d, there is a comparison between LOI_550–950_ and the sum of calcium carbonate and organic matter (calculated according to the formula in the figure text). Since that formula overestimates the fraction of other elements compared to carbon the amount of organic matter in GLD will be overestimated. Still the sum of calcium carbonate and organic matter still indicates calcite and organic matter as major components in GLD. By also adding NaOH and Mg(OH)_2_ to calcite and organic matter content (Fig. 5), most samples will reach a level of between 90 and 110%, indicating that these actually are the major components in GLD. This is in opposition to the general belief among process engineers and operating staff at the participating mill that GLD consists of mainly non-process elements, a small amount of remaining cooking chemicals, and a very small to no amount of organic material.

Samples with the lowest sum in Fig. 5 are Domsjö, Munksund, and Östrand. Domsjö is a biorefinery mill that uses sulfite as a cooking chemical and thus has a higher total sulfur content, 4.8–6.4%. The higher sulfur content as well as considering that not all sodium can be supposed to be in the form of NaOH can in part explain the low sum. Munksund has a very high efficiency when it comes to the recovery process, and is the mill that has the highest amount of processed wood per unit of GLD. This, in turn, means that the non-process elements are enriched in Munksund GLD as the dilution effect from organic content and process chemicals is lower in comparison to other mills (and Munksund does not use lime mud). Östrand on the other hand has, in general, noticeably higher concentrations of Al, Fe, Mn, and to some extent Si. During this study, the Östrand mill was rebuilt in several stages with the last two samples collected after works have been completed. It is likely that these higher amounts of major elements are due to a gradual break-in of new process equipment as the levels do decrease somewhat in the last two samples.

## pH and electrical conductivity

Both pH and electrical conductivity are high (Tables 7 and 8). pH is similar to what has been reported by others (Jia et al. 2017; Nurmesniemi et al. 2005), indicating equilibrium with solid Ca(OH)_2_ generating a pH of 12.45. Even higher pH usually indicates the presence of remaining NaOH in the solid residue, indicating poor washing. Electrical conductivity indicates that GLD generally contains large amounts of soluble salts that are easily washed out. Two mills, Vallvik and Skutskär, stand out as having much lower electrical conductivity, in general, compared to the other mills.

**Table 7:** pH values of samples

pH						
Mill	Spring 17	Fall 17	Spring 18	Fall 18	Spring 19	Average
Aspa	13.0	12.1	12.4	9.00	12.7	11.9
Billingsfors	13.4	12.6	12.7	12.7	12.7	12.8
Domsjö	No sample	12.5	12.7	12.8	12.6	12.6
Frövi	11.9	12.3	10.7	11.2	11.1	11.5
Gruvön	12.8	12.6	12.6	12.7	12.6	12.7
Gävle	10.9	12.2	11.5	9.82	12.4	11.4
Iggesund	12.5	12.5	12.6	12.6	12.6	12.5
Karlsborg	13.8	12.6	12.8	12.7	12.4	12.9
Munksund	13.2	9.22	11.7	12.6	11.5	11.7
Obbola	10.3	10.2	11.4	10.1	10.7	10.5
Piteå	No sample	11.7	10.6	12.6	12.5	11.8
Skoghall	No sample	11.9	11.8	10.9	10.2	11.2
Skutskär	12.0	No sample	No sample	No sample	No sample	12.0
Vallvik	10.3	12.3	11.9	12.1	11.6	11.6
Väja	13.8	No sample	No sample	12.1	No sample	12.9
Östrand	11.3	11.1	12.6	12.0	12.1	11.8

The value for Fall 17 for Domsjö is the average of both samples

**Table 8:** Electrical conductivity (mS/cm) values of samples

Electrical conductivity (mS/cm)						
Mill	Spring 17	Fall 17	Spring 18	Fall 18	Spring 19	Average
Aspa	31.0	35.0	38.1	27.6	50.3	36.4
Billingsfors	18.1	66.9	72.5	59.6	81.2	59.7
Domsjö	No sample	41.1 ± 4.35	43.0	56.9	46.8	45.8
Frövi	12.1	26.4	24.4	5.53	25.1	18.7
Gruvön	31.1	42.5	43.7	44.1	46.6	41.6
Gävle	11.2	14.8	8.7	3.82	29.2	13.6
Iggesund	18.4	28.1	26.5	25.0	31.0	25.8
Karlsborg	46.0	36.7	59.6	29.7	45.7	43.5
Munksund	9.8	7.05	20.1	43.9	21.0	20.4
Obbola	7.8	8.01	6.38	8.43	16.1	9.35
Piteå	No sample	9.71	12.8	18.0	32.2	18.2
Skoghall	No sample	57.9	28	6.96	28.6	30.4
Skutskär	5.7	No sample	No sample	No sample	No sample	5.70
Vallvik	1.87	20.5	8.8	0.14	3.39	6.94
Väja	21.6	No sample	No sample	35.1	No sample	28.4
Östrand	13.9	14.9	53.1	13.7	17.9	22.7

The value for Fall 17 for Domsjö is the average of both samples

### Elemental analysis

**Fig. 6:**
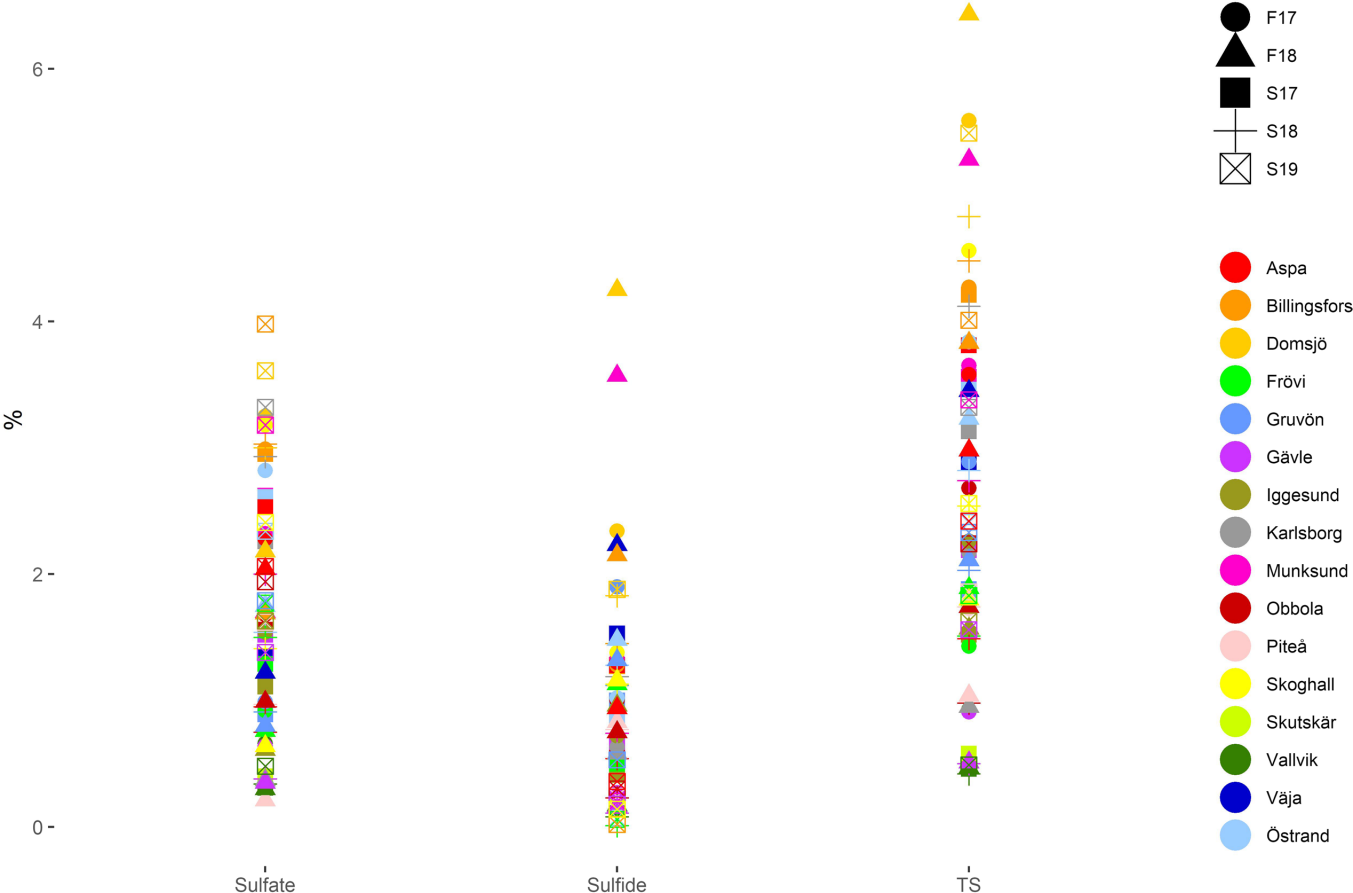
Concentrations (in weight %) of sulfate-S (measured), sulfide-S (calculated), and total sulfur (TS, measured) in all samples

Total sulfur content varies from < 1 to > 6% (Fig. 6). In general, samples contain less sulfide-S than sulfate-S, but the opposite does occur. Total sulfur content is in range with what others have reported (Jia et al. 2017, 2019; Golmaei 2018). The origin of sulfur is the Na_2_SO_4_ in the cooking chemicals that is not recovered during the chemical recovery of green liquor, as a 100% chemical recovery rate is not practically possible. It can be seen that mills that use lime mud during the separation of green liquor and GLD have lower sulfide-S concentrations, and thus also lower total sulfur concentration. This is probably due to Ca-carbonates in the lime mud reacting with sulfide-S and precipitating gypsum (CaSO_4_).

**Fig. 7:**
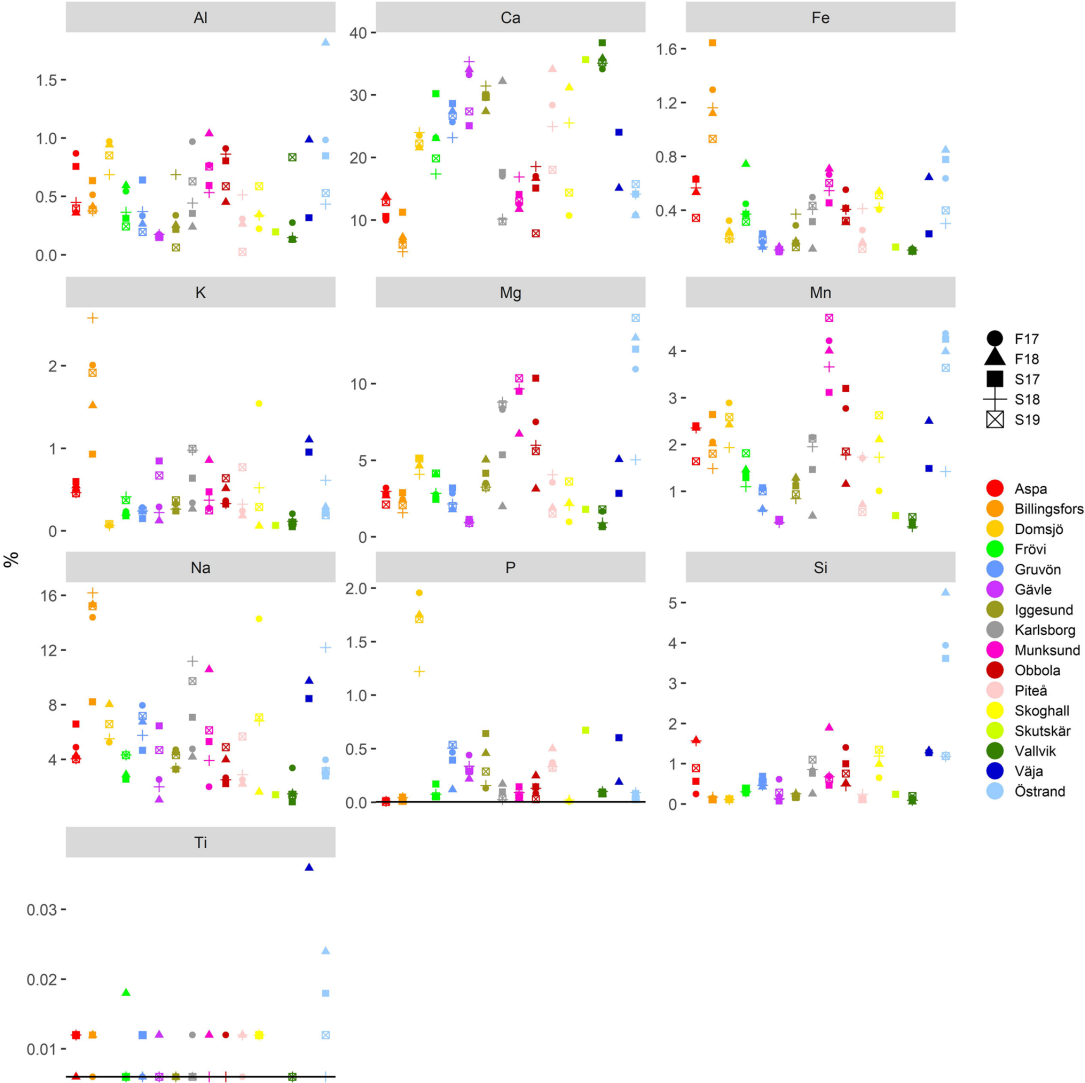
Concentrations (%) of major elements in all samples. The black line represents the detection limit; if no samples are below the detection limit, no detection limit line is present in the figure

Phosphorus concentrations (Fig. 7) are low (< 0.7%) in GLD from all mills except Domsjö (1.66 ± 0.31%) and are in line with earlier studies (see Table 1, Table 2, and Table 3 in supplementary data for references). Domsjö is a biorefinery mill with a sulfide cooking process and has higher phosphorus concentrations (more in line with concentrations reported by Martínez-Lage et al. (2016)). Phosphorus is derived solely from wood and the results indicate that the type of cooking process affects the fate and concentration of phosphorus in GLD. In the sulfide cooking process, phosphorus becomes a residual element ending up in the GLD. In the sulfate (kraft) cooking process, phosphorus is either volatilized during the black liquor incineration or diverted into the pulp during cooking.

Aluminum concentrations (Fig. 7) are generally at or below 1%, with one sample having higher concentration and similar values reported by others (Bandarra et al. 2019; Golmaei 2018; Jia et al. 2019; Martínez-Lage et al. 2016; see Table 1, Table 2, and Table 3 in supplementary data). However, concentrations are lower than those reported by Jia et al. (2017), Mäkitalo et al. (2014), Mäkitalo et al. (2016), Novais et al. (2018), and Mäkelä et al. (2016), but slightly higher than those reported by Cabral et al. (2008). In comparison to the sampling years reported in other studies, those in this study seem to indicate a trend where the aluminum concentrations in GLD decrease with time. Proposed reasons for this trend are changes in the chemical composition of lime mud, changes in the process line, or changes in analytical methods but further studies are needed to verify this trend and reasons behind it. In one study, the presence of aluminum in the form of berlinite (Al(PO)_4_) was found by SEM in one sample from Obbola (Hamberg et al. 2013).

**Fig. 8:**
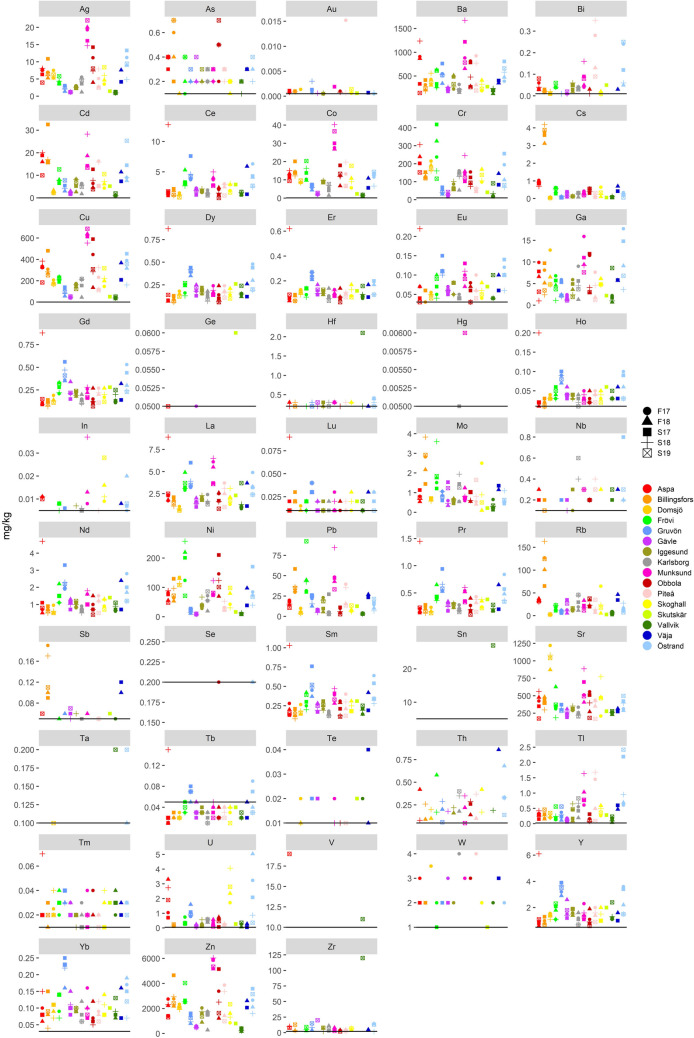
Concentrations (mg/kg d.w.) of trace elements in all samples. If no samples are below the detection limit no detection limit line is present in the figure

As expected, calcium is the most abundant element followed by sodium and magnesium (Fig. 7). Calcium has a very strong correlation with inorganic carbon (TIC) while magnesium is strongly correlated with manganese. In this study, calcium, sodium, and magnesium have similar concentrations as reported by others (Table 1, Table 2, and Table 3 in supplementary data); however the range reported, both by this study as well as others, is large for all three elements. Several researchers (Cabral et al. 2008; Mäkitalo et al. 2014; Pöykiö et al. 2006; Pérez-López et al. 2011; Sebogodi et al. 2020; Taylor and McGuffie 2007; Tran and Vakkilainnen 2008) have confirmed the presence of crystalline calcite by means of SEM microscopy and calcite was the only mineral present in all studies whereas other minerals varied depending on sample and study. Calcium has also been found in the form of gibbsite (CaSO_4_ × 2H_2_O) (Machado Martins et al. 2007), and calcium and sodium have also been found in the form of pirssonite (Na_2_Ca(CO)_3_ × 2H_2_O) (Taylor and McGuffie 2007; Manskinen et al. 2011; Stahre 2012; Hamberg et al. 2013). Burkeite (Na_6_(CO_3_)(SO_4_)_2_) was found by Hamberg et al. (2013) in GLD from Domsjö. Calcium and magnesium have been reported to have been found in the form of brucite (Mg(OH)_2_) (Mäkitalo et al. 2014; Hamberg et al. 2013) and Ca-Mg-carbonate (Machado Martins et al. 2007). Sodium, potassium, and rubidium have a very strong correlation with each other. Potassium (Fig. 7) and rubidium (Fig. 8) have generally similar concentrations between the mills whereas the variation of the other elements is large but in range with what others have reported (Table 1, Table 2, and Table 3 in supplementary data). Silicon and titanium concentrations (Fig. 7) are low in all mills except one, Östrand. Concentrations of titanium are in range with other reported values (Table 1, Table 2, and Table 3 in supplementary data) except for values reported by Mäkelä et al. (2016) as their values are higher than values in this study. Silicon concentrations are lower than those reported by Jia et al. (2017), but higher than what is reported by Mäkelä et al. (2016), and otherwise in range with concentrations reported by other references (Table 1, Table 2, and Table 3 in supplementary data). For manganese values reported in this study (Fig. 7) are in range with values reported from other Scandinavian mills (with the only exception being values reported by Mahmoudkhani et al. (2004)), but higher than those reported from non-Scandinavian mills (Table 1, Table 2, and Table 3 in supplementary data). Manganese is likely mostly derived from the cooked wood as lime mud used by the mills in this study generally has low concentrations (< 300 mg/kg dw) of manganese. The wood used in Sweden and Finland is often the same species and can sometimes come from areas that are geographically close, whereas mills in Portugal and Spain use different species of wood. One mineralogical study (Hamberg et al. 2013) has shown the presence of manganite (MnO(OH)) in GLD from Obbola. Sodium is almost solely derived from the cooking chemicals that remain in the dregs after washing as no mill has a 100% recovery rate for the spent cooking chemicals. Magnesium is most likely derived from wood while potassium is mainly derived from impurities in the cooking chemicals but in part also from the wood. Concentrations of potassium and magnesium in the lime mud used by the mills in this study are in general 10 times lower than those in the GLD.

Cs, Fe, Sb, Sr, and partly Mo have generally similar concentrations in GLD from all mills except for Billingsfors (Figs. 7 and 8). Billingsfors is one of two mills that do not wash out the last remaining green liquor from the dregs before dewatering. This indicates that these elements are water soluble and follow the wash water instead of remaining in the GLD. Cesium, molybdenum, antimony, and strontium are most likely solely derived from the wood used, and in the case of cesium, there might be a substantial contribution of Cs-137 to the total cesium concentration from the Chernobyl accident. Sweden and in particular the region around Uppsala, Gävle, and Västerbotten received high to very high fallout. McGee et al. (2000) noted 2370 Bq/m^2^ of Cs-137 in *Picea abies*, and 957 Bq/m^2^ in *Pinus sylvestris* in samples taken in a spruce forest in central Sweden*.* Iron on the other hand can have several sources. While the wood used is a likely main source, iron can also come from the water used for washing and dissolved from the equipment (mainly stainless steel). Jamborite ((Ni^2+^, Ni^3+^, Co, Fe^2+^, Fe^3+^)(OH)_2_(OH, S, H_2_O)) has been found by SEM in GLD samples from Obbola (Hamberg et al. 2013). Strontium concentrations in this study are lower than what Martínez-Lage et al. (2016) have reported, while iron concentrations are consistent with what others have reported (Table 1, Table 2, and Table 3 in supplementary data).

Arsenic concentrations (Fig. 8) are lower than 0.7 mg/kg dw with approximately one third of the samples below detection limit; this is in range with what several others have reported (Table 1, Table 2, and Table 3 in supplementary data) but lower than what Jia et al. (2017) reported. Cadmium is generally below 20 mg/kg dw but three samples have higher concentrations (Billingsfors, Munksund, and Östrand). Cadmium concentrations are somewhat lower than reported by Golmaei (2018), higher than reported by Mäkitalo et al. (2014), significantly higher than reported by Sebogodi et al. (2020), but in range with those reported by Cabral et al. (2008), Jia et al. (2019), and Mäkelä et al. (2016). Lead concentrations are generally < 50 mg/kg dw with samples from Billingsfors, Frövi, and Munksund exceeding that. Lead concentrations are in range with what is reported by some other references (Table 1, Table 2, and Table 3 in supplementary data), lower than what is reported by Cabral et al. (2008) but higher than reported in Mäkitalo et al. (2014) and Mäkitalo et al. (2016). Lead, arsenic, and cadmium are derived mainly from wood. Several researchers have shown that the European red pine, *Pinus sylvestris*, is sensitive to trace element pollution due to uptake of cadmium and lead (Chudzińska et al. 2014; Kandziora-Ciupa et al. 2016; King et al. 1984; Österås 2004; Österås and Greger 2006). Studies done by King et al. (1984) showed that both spruce and pine can also bioaccumulate arsenic. While bioavailable cadmium and lead content in Swedish soil has decreased in the last two centuries due to regulation (Hunová et al. 2023), during the latter half of the 1900th century atmospheric deposition from air pollution and acid deposition was significant (Österås 2004). The average life span of a tree felled in Sweden is 45–120 years, depending on species and geographical location, meaning that most trees felled and processed today in the paper and pulp industry has grown most of their life span during times of high dry deposition of cadmium and lead.

Ba, Cr, Cu, Ni, and Zn generally occur in high concentrations that varies considerably between mills, and zinc is the most abundant trace element (Fig. 8). The same four mills (Munksund, Obbola, Billingsfors, and Östrand) reoccur as having the highest concentrations of these elements. Cobalt, copper, and zinc have a very strong correlation to each other together with silver (Fig. 8). Barium concentrations are generally lower than reported by other references while copper concentrations are in the same range as reported earlier (Table 1, Table 2, and Table 3 in supplementary data). For chromium, concentrations are in range with what is reported by other references except for Sebogodi et al. (2020), which has significantly higher reported concentrations compared to this study. Nickel concentrations are in the same range as reported by Jia et al. (2019), Mäkelä et al. (2016), and Golmaei (2018), higher than those reported by Mäkitalo et al. (2014), Mäkitalo et al. (2016), and Jia et al. (2017), while being significantly lower than what is reported by Cabral et al. (2008), Pöykiö et al. (2006), and Sebogodi et al. (2020). Zinc concentrations in this study are generally higher than those reported by several references (Table 1, Table 2, and Table 3 in supplementary data), but at the same time in range with what other references have reported, and also lower than those reported by Jia et al. (2019). High concentrations of these trace elements can be problematic from a remediation point of view. The goal is to reduce trace metal concentrations in the acid mine drainage and if the remediant, in this case GLD, adds a load to the trace metal concentration it potentially defeats the purpose of using GLD as a remediant.

Cobalt concentrations are generally at or below 20 mg/kg dw except for samples from Munksund that are higher (Fig. 8). Cobalt concentrations are higher than reported in Mäkitalo et al. (2014) and Mäkitalo et al. (2016), lower than reported by Cabral et al. (2008) and otherwise in range with other studies (Table 1, Table 2, and Table 3 in supplementary data). Gold is near or below the detection limit for all samples except the sample from the Piteå mill in spring 2017 (Fig. 8). For yttrium, the concentrations are generally less than 6 mg/kg dw, with Gruvön and Östrand having somewhat higher concentrations than the rest of the samples (Fig. 8). Gallium is present in all samples, but the maximum concentration detected was less than 18 mg/kg dw, with Munksund and Östrand having the highest concentrations (Fig. 8). Thallium concentrations are generally less than 1 mg/kg dw with a few samples from Munksund, Piteå, and Östrand exceeding that (Fig. 8). Uranium concentrations are generally less than 2 mg/kg dw with a few samples from Aspa, Skoghall, and Östrand mill exceeding that (Fig. 8). Bismuth concentrations are generally less than 0.1 mg/kg dw with 2 samples from Piteå and Östrand mill each exceeding 0.1 mg/kg dw (Fig. 8).

Ge, Hg, In, Se, Sn, Ta, Te, Nb, Sb, and V were only detected in very few samples, with samples from spring 2019 having most samples above the detection limit (Fig. 8). Indium and thorium (most commonly present in samples from spring and fall 2018), hafnium and zirconium (only one sample exceeding 25 mg/kg dw) are present in less than half of the samples and concentrations remain around detection limit (Fig. 8). In general, REE (rare earth element) concentrations in this study are higher than concentrations reported by Golmaei et al. (2018) (Table 4 in supplementary data). Tungsten is only present above the detection limit in samples from 2017 (Fig. 8). This might be an analytical effect where tungsten is underestimated in the other samples. Samples from 2017 were analyzed in the same, first batch, whereas the other sampling series were analyzed in separate batches. The lanthanides have a high correlation with each other, with one sample from Gruvön mill, collected during spring 2017, having the highest concentrations for several of the elements (Fig. 8). In general, concentrations are low or below the detection limit except for samples from Gruvön, followed by samples from Östrand, that have higher concentrations than the rest of the samples, who otherwise have similar concentrations.

Gruvön, Östrand, Billingsfors, and Munksund stand out as samples from these mills most often show the highest concentrations of trace elements and clear groupings of elements. Gruvön is the mill that has the highest concentrations of the lanthanoids with Östrand being the mill with the second highest concentrations. For Gruvön this may be a result of their cooking process. Gruvön is mainly a sulfate kraft mill but instead of using white liquor, they use red liquor for cooking, making the actual cooking properties like sulfide cooking (but their recovery line is regarded as a pure sulfate recovery process). Munksund (together with Gruvön) stands out as having generally higher concentrations of Ag, Ba, Co, Cu, Mn, Zn, and La (Fig. 8). It is also one of the mills that have the highest cadmium concentrations together with Aspa and Billingsfors. This is most likely due to the fact that Munksund, together with Obbola, is the mill that has the highest concentrations of non-process elements as they have the highest ratio of m^3^fub wood processed/tonne dry GLD (Table 2). Munksund processes around 2300 m^3^fub/tonne dry weight GLD, which is 2 to 4 times as much as the other mills. Obbola processes around 1950 m^3^fub/tonne dry weight GLD but generally has newer equipment. This indicates that as the amount of wood processed increases, so does the concentration of non-process elements in the GLD, but factors such as type of wood/ratio of wood types and process-specific details also have a noticeable impact. Mechanical separation methods have been shown to create physical separation of Cd, Ni, Pb, and Zn (Kinnarinen et al. 2018; Golmaei et al. 2018), and the rate and temperature of filtration when separating dregs from green liquor by cake filtration can affect the concentration of boron, potassium, and sodium in the filter cake (Golmaei et al. 2017).

For Billingsfors it is most likely the fact that this is one of two mills in the study that do not wash the remaining green liquor after the separation of green liquor and GLD, therefore increasing the amount of water-soluble elements in the dregs. For Östrand chromium, gallium, silicon, thallium, and uranium concentrations first increase, and then decrease in correspondence with the mill’s expansion and gradual transfer to a new state-of-the-art equipment. The gradual replacement of the mill’s equipment can also be the reason for the detectable concentrations of lanthanoids. However, the specific details of the equipment change have not been provided. The lanthanides concentrations in Östrand are very similar between samples. This indicates that the origin of these elements is one and the same, and the lowest concentrations are in the latest samples where the equipment has been in use for a while.

### Comparison with Swedish till

To better discern if an element is of environmental concern, elemental concentrations in this study were compared to elemental concentrations in Swedish till, the most common soil covering about 75% of the country (SGU 2014a). The Geological Survey of Sweden (SGU) has extensive records of geochemical analyses of till (SGU 2014a).

A comparison was performed between the average element concentrations in GLD and background concentrations of elements in till (Tables 9 and 10). Background concentrations were defined as the 95th percentile according to Ander et al. (2013). Till were sampled in the C-horizon in the soil at a depth of at least 0.8 m and digested using aqua regia digestion. For Al, Fe, Ti, As, Au, Bi, Ce, Co, Cs, Dy, Er, Eu, Ga, Gd, Hf, Ho, La, Lu, Mo, Nb, Nd, Pr, Sb, Sm, Tb, Te, Th, Tm, U, V, Y, Yb, and Zr the average concentration in GLD was well within the 90th percentile range for till, indicating that these elements are of low concern as the background level is higher.

**Table 9:** Concentrations (mg/kg dw) of major elements in GLD and Swedish till (SGU 2014a) as well as the GLD/till ratio, and elements in the two most commonly used wood types in Swedish paper and pulping industry (Andersson 1977)

	GLD *Average*	Till *Background level*	Ratio *GLD/till*	European red pine (*Pinus sylvestris*)	Norway spruce (*Picea abies*)
Al	5100 ± 3100	23,900	0.21	-	-
Ca	210,700 ± 90,200	8110	26.0	-	-
Fe	4100 ± 3100	36,200	0.11	-	-
K	4800 ± 4800	4320	1.11	-	-
Mg	42,800 ± 32,300	9450	4.53	-	-
Mn	17,900 ± 11,400	700	25.6	12	194
Na	54,600 ± 36,200	688	79.4	-	-
P	2900 ± 4400	1290	2.21	-	-
Si	6800 ± 8800	-	-	-	-
Ti	100 ± 100	2940	0.04	-	-

A ratio higher than 1 indicates enrichment in the GLD

**Table 10:** Concentrations (mg/kg dw) of minor elements in GLD and Swedish till (SGU 2014a) as well as the GLD/till ratio, and elements in the two most commonly used wood types in Swedish paper and pulping industry (Andersson 1977)

	GLD *Average*	Till *Background level*	Ratio *GLD/till*	European red pine (*Pinus sylvestris*)	Norway spruce (*Picea abies*)
Ag	5.89 ± 4.66	0.101	58.3	-	-
As	0.304 ± 0.15	13.8	0.022	-	-
Au	0.0015 ± 0.003	0.004	0.379	-	-
Ba	450 ± 293	121	3.71	-	-
Bi	0.060 ± 0.071	0.493	0.122	-	-
Cd	8.73 ± 6.73	0.206	42.4	0.1	0.34
Ce	2.58 ± 1.83	148	0.017	-	-
Co	10.7 ± 7.78	16.7	0.639	0.08	< 0.05
Cr	121 ± 79.5	56.0	2.17	2.3	1.9
Cs	0.528 ± 0.910	3.95	0.134	-	-
Cu	233 ± 161	49.9	4.67	5.6	3.6
Dy	0.195 ± 0.125	6.15	0.032	-	-
Er	0.118 ± 0.080	3.42	0.035	-	-
Eu	0.071 ± 0.034	1.42	0.050	-	-
Ga	5.51 ± 3.83	8.14	0.677	-	-
Gd	0.221 ± 0.130	7.56	0.029	-	-
Ge	0.053 ± 0.005	-	-	-	-
Hf	0.329 ± 0.346	0.804	0.409	-	-
Hg	0.0055 ± 0.0005	-	-	0.01	0.02
Ho	0.041 ± 0.028	1.21	0.034	-	-
In	0.011 ± 0.007	-	-	-	-
La	2.50 ± 1.54	58.4	0.043	-	-
Lu	0.019 ± 0.01	0.447	0.043	-	-
Mo	0.998 ± 0.741	2.37	0.421	-	-
Nb	0.277 ± 0.148	6.67	0.042	-	-
Nd	1.19 ± 0.713	49.9	0.024	-	-
Ni	70.6 ± 54.9	41.2	1.71	1.2	0.72
Pb	19.4 ± 17.9	25.6	0.757	2.2	2.8
Pr	0.340 ± 0.216	13.7	0.025	-	-
Rb	25.6 ± 29.2	43.9	0.583	-	-
S	25,700 ± 14,200	217	118	-	-
Sb	0.080 ± 0.039	0.588	0.135	-	-
Se	0.200 ± 2.8 × 10^−17^	-	-	-	-
Sm	0.277 ± 0.160	9.36	0.030	-	-
Sn	27	2.36	-^a^	-	-
Sr	412 ± 232	57.3	7.18	-	-
Ta	0.150 ± 0.050	-	-	-	-
Tb	0.035 ± 0.022	1.09	0.032	-	-
Te	0.017 ± 0.008	0.056	0.302	-	-
Th	0.268 ± 0.176	19.5	0.014	-	-
Tl	0.452 ± 0.465	0.486	0.930	-	-
Tm	0.026 ± 0.011	0.472	0.054	-	-
U	0.827 ± 1.061	5.61	0.147	-	-
V	15.0 ± 4.0	71.4	0.210	-	-
W	2.45 ± 0.84	1.08	2.27	-	-
Y	1.66 ± 0.94	32.3	0.052	-	-
Yb	0.109 ± 0.044	3.13	0.035	-	-
Zn	2130 ± 1420	90.3	23.6	44	40
Zr	11.6 ± 22.1	28.2	0.412	-	-

A ratio higher than 1 indicates enrichment in the GLD

^a^As tin was only detected in one GLD sample no ratio was calculated

Elements that are typically enriched in GLD in comparison with till are generally elements where there is an uptake of elements in trees (Perelman 1989; Baltrėnaitė et al. 2012). In turn, it can be inferred that elements that are depleted in GLD are most likely elements that are generally not taken up by trees.

K, Pb, Rb, and Tl have similar concentrations in GLD as in till, meaning that these elements are of minor concern in general, but it is prudent to be mindful of the risk regarding rarely occurring extreme concentrations for these elements in GLD.

Generally, Ca, Mg, Mn, Na, P, S, Ag, Ba, Cd, Cr, Cu, Ni, Sn, Sr, W, and Zn are all elements with concentrations in GLD above the 95th percentile in till. This does not mean that all these elements are of concern. The major elements Ca, Mg, and P are typically not considered an environmental problem. For example, liming soils is a common practice in agriculture and the lime used mainly consists of CaCO_3_ with addition of dolomite minerals to elevate magnesium concentrations, and phosphorous is one of the most important elements in fertilizers. Sodium concentrations in GLD are generally very high, and this may be problematic from an environmental standpoint. It has been found that the usage of de-icing salts, which mainly contain NaCl, tends to increase trace element mobility in water. This effect has been attributed to mainly cation exchange (Bäckström et al. 2004; Löfgren 2001; Norrström and Bergstedt 2001), but studies done by Pontoni et al. (2019) showed that when sodium concentration was high the mobilization of trace metals decreased again. However, these studies did not include the extremely high sodium concentrations that are present in GLD. Seen in the context of remediation of acid mining waste with alkaline GLD, the possible effect of sodium increasing trace metal mobility is most likely of little concern. Although potassium concentrations in this study are greater than the 95th percentile of till, they are not higher than potassium concentrations in other soils. For example, loess soils in southern Poland have 1.6–2.0% potassium (Drewnik et al. 2014). Manganese is an essential element but can become toxic when present in high concentrations. However, many plants can avoid manganese toxicity by different mechanisms, in particular in the presence of high phosphorous concentrations (Zemunik et al. 2020), meaning that high manganese concentrations in substrate are not necessarily an environmental problem but further study is necessary. Sulfur and trace elements, in particular sulfur and the base metals, are on the other hand of concern. High sulfur (mainly in the form of sulfate-S) and base metals concentration in the ARD effluent together with low pH are the main environmental problems that GLD is to alleviate. However, as GLD is not a naturally formed material it is necessary to do leaching tests as a high concentration in solid GLD does not necessarily mean that the element is leached out. For example, Jia et al. (2017), Jia et al. (2019), and Mäkitalo et al. (2014) had similar zinc concentrations in their studied GLD, but when they performed batch leaching tests the leached concentrations were less than 0.1 mg/kg for Jia et al. (2017) and Mäkitalo et al. (2014), and less than 9 mg/kg for Jia et al. (2019). If the element is immobile and does not leach, high elemental concentration in the solids is of minor concern. Further indication that high element concentrations do not necessarily pose a problem is found by Österås et al. (2005) and Zambano et al. (2010). Österås et al. (2005) found that the addition of GLD to wood ash, when using it as a treatment for soil nutrient depletion in the forest industry, increased the performance of the ash and lowered the concentration of Zn, Cu, and Cd in the tree stems. Zambano et al. (2010) found that the addition of GLD to Kraft mill sludge increased composting performance without affecting microbial biodiversity.

### PCA analysis

**Fig. 9:**
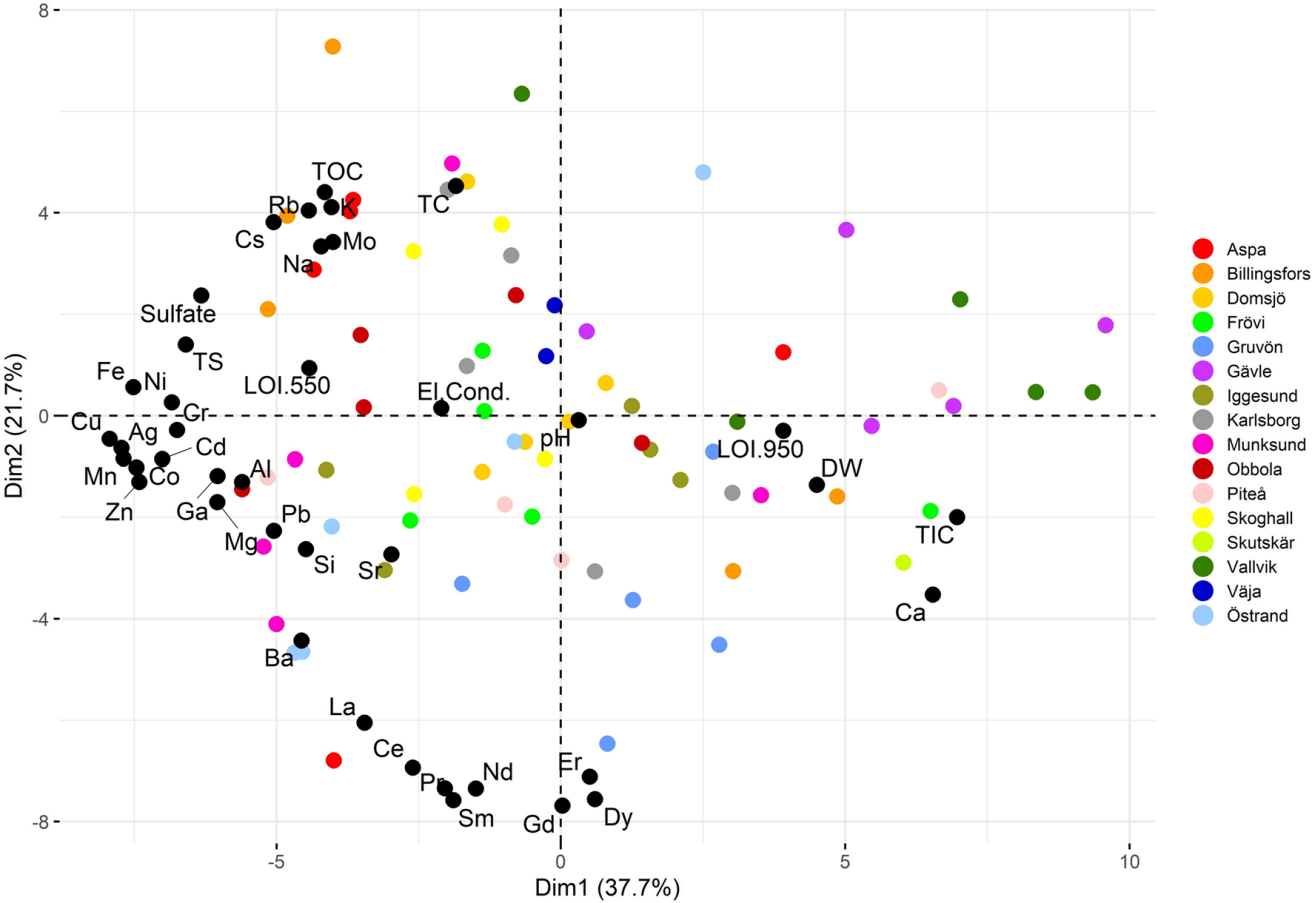
The PCA results plotted as a biplot. Loadings are represented as black dots and scores as dots where each mill has a separate color; the first two components explain 59.4%

PC (principal component) 1 accounts for 37.7% of the sample similarity, and together with PC 2 they represent 59.4% (Fig. 9). In total, 90% of the sample similarity is explained by the first 9 components, meaning that sample heterogeneity is large. Grouping of the mills is weak further indicating that there is a large heterogeneity in GLD composition.

The Östrand samples appear to have the largest heterogeneity within a mill’s samples; this is expected as the Östrand mill has done a large-scale remodeling of the process line over the sampling period. Mills that have GLD containing lime mud tend to plot to the right (Dim2 > 0) while mills with non-lime mud-containing GLD tend to plot to the left but there is no clear grouping. Calcium, TIC, DW, and LOI_950_ group together and these parameters are one of the most influential factors for sample similarity. This is expected as calcium is very heavily dependent on TIC as almost all calcium in GLD originate from the lime mud carbonates, and the biggest influence on LOI_950_ is the volatilization of carbonates into CO_2_. This group is distinct and stands out from the other groups the most when regarding the first component. That Na, K, Rb, and Cs group together is expected as the major elements sodium and potassium are almost entirely dependent on the recovering effect of the cooking chemicals, and the trace elements rubidium and cesium follow the major elements. However, having molybdenum, TOC, and partly TC also in this group is unexpected and the connection between these elements is currently not known. It is possible that this is a coincidence, but further study is necessary. LOI_550_ plots closer to the sulfur species than TOC in this PCA. It is common to equate LOI_550_ with TOC as organic material burns off at 550 °C, but these results indicate that the sulfur species are more closely related to LOI_550_ than the organic matter content. This means that when a sample has a high sulfur content, LOI_550_ cannot be used as the sole determinant of organic matter as the sulfur combusts and the mass lost when ashing at 550 °C contains both sulfur species as well as organic carbon species. The rare earth elements have a clear grouping as do the base metals, but the base metals seem to have a larger impact on sample differentiation as PC 2 has a low importance. Grouped together with the base metals we also see Al, Fe, Mn, Mg, and Si. The REE elements are geochemically similar and that is probably the reason for their grouping. The origin of most of the elements in the base metal group is the wood used and that explains their grouping.

## Conclusion

GLD is a heterogeneous material, both between different mills but also between different sampling occasions within a mill to a lesser degree. There are, however, some common characteristics for all GLD. In general, GLD consists mainly of organic carbon, inorganic carbon (carbonates), Ca, Mg, Na, and S. Organic carbon originates from the wood itself, and while all remaining organic material is supposed to be combusted in the recovery boiler, some still remains. Inorganic carbon and calcium originate from the last step before disposal, where some mills use lime mud during the dewatering process of GLD. Magnesium originates both from the wood itself and from being a contaminant in the white liquor used for pulping, and finally, sodium and sulfur are remaining cooking chemicals not fully washed out of the GLD before disposal. While mineralogical studies have been able to identify some minerals such as calcite, GLD mainly consists of poorly crystalline solids.

Process-specific details at a mill can have a large impact on GLD in terms of dry matter content, loss of ignition, calorific value, and elemental composition. In general, a GLD sample either has several parameters deviating from the population or none, and most samples are similar to each other. When looking at using GLD on a larger scale for remediation purposes, the differences between samples generally do not pose a significant problem, as all samples belong to the same population since no clear grouping occurs.

It is reasonable to conclude that it is not necessary to analyze and study Al, Fe, Ti, As, Au, Bi, Ce, Cs, Dy, Er, Eu, Gd, Ho, La, Lu, Nb, Nd, Pr, Sb, Sm, Tb, Te, Th, Tm, U, V, Y, Yb, and Zr for every single case of using GLD as a remediant as the concentrations of these elements are well within the range for background levels in Swedish till. When comparing the elemental concentration in this study to the elemental concentration in Swedish till, most trace elements and some major elements are of no concern. Elements of most concern are sulfur species, Cd, Cr, Cu, Ni, and Zn as high concentrations of these elements in GLD can pose a problem when using GLD for mine site remediation purposes, as the mine sites often already have high concentrations of these elements in the effluent. The high total concentrations in themselves are not a problem if the elements are immobile and do not leach out of the GLD.

From the viewpoint of remediation of acidic sulfidic mine sites, high organic content in a remediant is beneficial as oxidation of organic material consumes oxygen and can therefore lower oxidation of sulfides and production of ARD. Thus, high TOC content in GLD is a good quality from a geochemical standpoint. However, Swedish regulations stipulate that materials containing > 4% organic content are not allowed to be landfilled, and currently, this regulation applies to the use of waste materials such as GLD for mine site remediation.

Overall, this study has found that as an initial assessment, GLD can be a good alternative to traditional materials for cost-efficient remediation of smaller orphaned mine sites. It is readily available in large quantities, has the qualities needed for remediation of acidic mine sites, and can often be relatively locally sourced near the mine site. The reuse of GLD for the remediation of mining sites is also congruent with the concept of circular economy. However, to fully categorize GLD as a feasible option for mining waste remediation leachability and long-term alkalinity properties needs to be evaluated.

### Supplementary Information

Below is the link to the electronic supplementary material.

Supplementary file1 (DOCX 119 KB)

Supplementary file2 (JPEG 272 KB)

## Data Availability

Datasets used in this study are available from the corresponding author upon reasonable request.
