# Supplementary material for: Chemical characterization of green liquor dregs from 16 Swedish pulp and paper mills between 2017 and 2019
Source: Environ Sci Pollut Res Int. 2024 Jul 3;31(32):45011–34. doi: 10.1007/s11356-024-34074-3 (PMC11254974; doi:10.1007/s11356-024-34074-3)
Supplement: Supplementary file 1 — Supplementary file1 (DOCX 119 KB) [file 11356_2024_34074_MOESM1_ESM.docx]

Supplementary data to *Chemical characterization of Green Liquor Dregs from 16 Swedish pulp and paper mills between 2017 and 2019*

Nanna Stahre^1^, Lotta Sartz^1,2^, Mattias Bäckström^1,2^

^1^Man-Technology-Environment Research Centre, Örebro University, 701 82 Örebro, Sweden

^2^Bergskraft Bergslagen AB, Södra Kungsvägen 49, 692 30 Kumla, Sweden


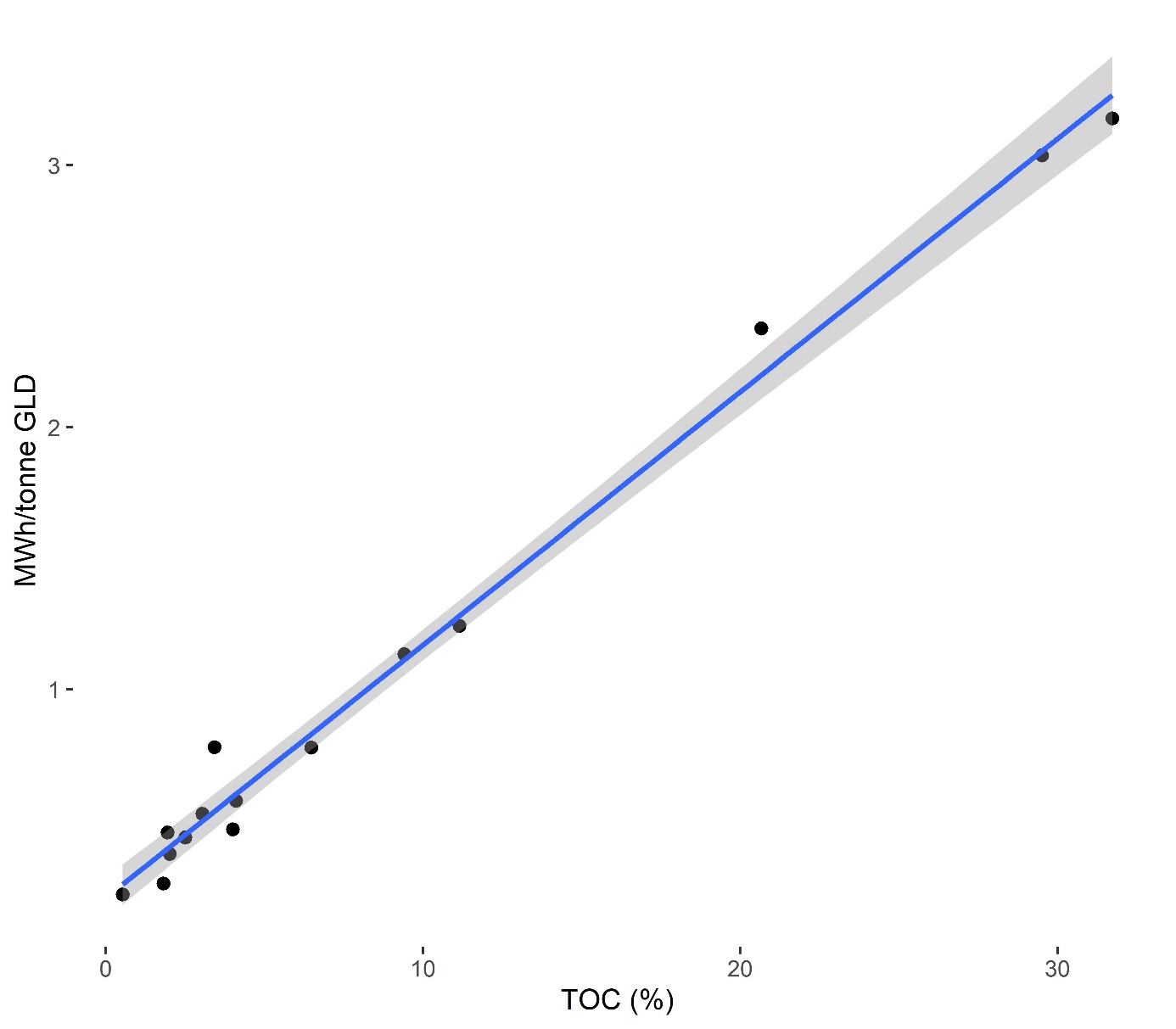


Fig. 1 TOC (%) plotted against calorific value (MWh/tonne) for all samples taken fall 2018 (R^2^ = 0.99)

Table 1 Concentrations of elements in GLD in this study compared to other sources (mg/kg dw), n=no of replicates

|  | **This study** | **Bandarra et al. (2019)** | **Cabral et al. (2008)** | **Golmaei (2018)** | **Golmaei (2018b)** | **Golmaei (2018c)** | **Jia et al. (2017)** |
| --- | --- | --- | --- | --- | --- | --- | --- |
| Country of origin | Sweden | Portugal | Portugal | Finland | Finland | Finland | Sweden |
| No mills participating | 16 | 1 | 1 | 10 | 1 | 1 | 1 |
| No of samples | 71 | 1 | 4 | 20 | 1 | 1 (n=1) | 1 (n=3) |
| Al | 5 100 ± 3 100 | 7 000 | 300 – 4 100 | 1 010 – 20 200 | - | - | 19 300 ± 2 700 |
| Ca | 210 700 ± 90 200 | 256 000 | 118 200 – 347 000 | 99 000 – 346 000 | 183 000 | - | 175 000 ± 4 000 |
| Fe | 4 100 ± 3 100 | 3 300 | 2 600 – 5 400 | 970 – 20 700 | 1 840 | - | 11 900 ± 2 800 |
| K | 4 800 ± 4 800 | 600 | 900 – 6 600 | 260 – 7 590 | 8 500 | - | 17 900 ± 1 600 |
| Mg | 42 800 ± 32 300 | 122 000 | 21 000 – 51 100 | 8 970 – 97 600 | 43 900 | - | 25 100 ± 1 700 |
| Mn | 17 900 ± 11 400 | 8 900 | 1 600 – 7 800 | 4 760 – 31 600 | 12 700 | - | 11 100 ± 500 |
| Na | 54 600 ± 36 200 | 72 500 | 29 800 – 144 400 | 6 360 – 107 000 | 105 000 | - | 40 700 ± 1 400 |
| P | 2 900 ± 4 400 | 1 200 | 900 – 2 100 | 600 – 4 920 | 2 610 | - | - |
| Si | 6 800 ± 8 800 | 15 300 | - | - | - | - | 72 100 ± 11 600 |
| Ti | 100 ± 100 | - | - | < 50 - 450 | - | 51.9 | - |
| As | 0.304 ± 0.15 | - | - | < 3 | - | - | 3.7 ± 0.3 |
| Ba | 450 ± 293 | 300 | - | 230 - 920 | - | - | 772 ± 28 |
| Cd | 8.73 ± 6.73 | - | 4.07 - 6.12 | 3.8 - 30 | 9.4 | - | 8.0 ± 0.5 |
| Co | 10.7 ± 7.78 | - | 18.7 - 25.1 | 3.1 - 29 | 7.7 | 8.84 | - |
| Cr | 121 ± 79.5 | - | 47.4 - 68.9 | 33 - 240 | 102 | - | 67.2 ± 0.4 |
| Cu | 232 ± 161 | - | 30.2 - 102.0 | 78 - 420 | 158 | - | 254 ± 163 |
| Hg | 0.0055 ± 0.0005 | - | - | - | - | - | <0.04 |
| Mo | 0.998 ± 0.74 | - | - | < 1.0 - 6.2 | - | - | 2.4 ± 0.4 |
| Ni | 1.19 ± 0.71 | - | 119.0 - 254.0 | 16 - 340 | 38 | - | 40.8 ± 0.6 |
| Pb | 19.4 ± 17.9 | - | 31.1 - 62.4 | 3.8 - 47 | 11 | 16.9 | 22.7 ± 3.4 |
| S | 25 700 ± 14 200 | 61 800 | - | 4 160 – 58 100 | - | - | 14 100 ± 611 |
| Sr | 412 ± 232 | - | - | - | - | - | - |
| V | 15 ± 4 | - | - | < 2 – 54 | - | - | - |
| Zn | 2 130 ± 1 420 | - | 48.8 - 258.0 | 620 – 5 790 | 1 560 | - | 1 450 ± 72 |

Table 2 Continuation of table of concentrations of elements in GLD as reported by other references (mg/kg dw), n=no of replicates, to be noted is that values presented in Mäkitalo et al 2016 are also presented in Jia et al 2019

|  | **This study** | **Jia et al. (2019)^a^** | **Mahmoudkhani et al. (2004)** | **Manskinen et al. (2011)** | **Martínez-Lage et al. (2016)** | **Mäkela et al. (2016)** | **Mäkitalo et al. (2014)** |
| --- | --- | --- | --- | --- | --- | --- | --- |
| Country of origin | Sweden | Sweden | Sweden | Finland | Not specified (likely Spain) | Sweden | Sweden |
| No of mills participating | 16 | 5 | 1 | 1 | 1 | 1 | 1 |
| Total no of samples | 71 | 7 (n=3) | 1 | 1 (composite) | 1 | 1 (compsite) | 4 (n=2) |
| Al | 5 100 ± 3 100 | 1 960 – 16 800 | 7 520 | 820 | 2 280 | 25 500 | 2 700 – 9 890 |
| Ca | 211 000 ± 90 200 | 67 800 – 295 000 | 87 900 | - | 330 000 | 175 000 | 217 000 – 317 000 |
| Fe | 4 100 ± 3 100 | 3 570 – 11 300 | 6 990 | 600 | 5 600 | 8 010 | 3 170 – 9 650 |
| K | 4 800 ± 4 800 | 830 – 21 600 | 14 700 | - | 2 320 | 1 400 | 1 910 – 3 570 |
| Mg | 42 800 ± 32 300 | 11 900 – 65 700 | 115 000 | - | 19 900 | 95 900 | 37 800 – 73 300 |
| Mn | 17 900 ± 11 400 | 9 140 – 38 300 | 39 600 | 4 150 | 3 180 | 35 100 | 8 350 – 17 500 |
| Na | 54 600 ± 36 200 | 15 600 – 203 000 | 87 500 | - | 25 200 | 19 800 | - |
| P | 2 900 ± 4 400 | - | 262 | - | 14 400 | 200 | 911 – 4 030 |
| Si | 6 800 ± 8 800 | 1 080 – 14 300 | 22 000 | 270 | 6 080 | - | 4.96 - 12.4 |
| Ti | 100 ± 100 | - | - | - | - | 420 | - |
| As | 0.304 ± 0.15 | < 0.1 - 0.80 | 12.2 | <3 | - | - | 0.172 - 0.474 |
| Ba | 450 ± 293 | - | 737 | 910 | - | 960 | - |
| Cd | 8.73 ± 6.73 | 2.62 - 25.7 | 38.6 | 3.8 | - | 11 | 3.54 - 8.65 |
| Co | 10.7 ± 7.78 | 3.04 - 19.4 | 12.6 | 8.0 | - | 19 | 3.04 - 5.77 |
| Cr | 121 ± 79.5 | 25 – 295 | 332 | 14.0 | - | 160 | 71.9 - 127 |
| Cu | 232 ± 161 | 117 – 532 | 266 | 61.0 | 184 | 330 | 101 - 224 |
| Hg | 0.0055 ± 0.0005 | < 0.05 | 0.08 | <0.04 | - | - | <0.04 |
| Mo | 0.998 ± 0.74 | 0.29 - 2.62 | - | <1.0 | - | - | - |
| Ni | 1.19 ± 0.71 | 22 - 255 | - | 14.0 | 118 | 80 | 29.3 - 60.8 |
| Pb | 19.4 ± 17.9 | 6.12 - 36.7 | 60.3 | 13.0 | - | 52 | 2.27 - 6.13 |
| S | 25 700 ± 14 200 | 11 800 – 47 800 | 51 400 | - | 5 610 | 31.2 | 7.88 - 18.7 |
| Sr | 412 ± 232 | - | 470 | - | - | - | - |
| V | 15 ± 4 | - | - | <50 | - | 3.4 | - |
| Zn | 2 130 ± 1 420 | 700 – 4 470 | - | 1 320 | 1 210 | 6 490 | 701 – 1 840 |

a) values presented in Mäkitalo et al 2016 are also presented in Jia et al 2019

Table 3 Continuation of table of concentrations of elements in GLD as reported by other references (mg/kg dw), n=no of replicates

|  | **This study** | **Mäkitalo et al. (2016)^a^** | **Novais et al. (2018)** | **Nurmesniemi et al. (2005)** | **Pöykiö et al. (2006)** | **Sebogodi et al. (2020)^b^** | **Zołnowski et al. 2019** |
| --- | --- | --- | --- | --- | --- | --- | --- |
| Country of origin | Sweden | Sweden | Portugal | Finland | Finland | South Africa | Poland |
| No of mills participating | 16 | 1 | 1 | 1 | 1 | 2 | 1 |
| Total no of samples | 71 | 1 (n=3) | 1 | 1 (composite) | 1 (n=3) | 2 | 1 |
| Al | 5 100 ± 3 100 | 2 710 ± 14 | 14 000 | - | - | 2 270 - 3 760 | - |
| Ca | 210 700 ± 90 200 | 317 000 ± 0 | 109 000 | 84 500 ± 1 400 | 84 500 | 101 000 – 198 000 | 242 400 |
| Fe | 4 100 ± 3 100 | 3 180 ± 14 | 4 760 | - | 4 500 | 3 530 - 4 650 | - |
| K | 4 800 ± 4 800 | 1 980 ± 99 | 7 390 | 1 700 ± 100 | 1 700 | 5 420 - 5 430 | 5 420 |
| Mg | 42 800 ± 32 300 | 38 100 ± 353 | 49 000 | 29 200 ± 900 | 29 700 | 16 400 - 39 600 | 41 200 |
| Mn | 17 900 ± 11 400 | 8 390 ± 57 | 6 350 | - | 10 300 | 7 760 - 9 550 | - |
| Na | 54 600 ± 36 200 | 20 950 ± 212 | 177 000 | 26 100 ± 1 200 | 26 100 | 71 100 - 153 000 | 57 600 |
| P | 2 900 ± 4 400 | 4 020 ± 21 | 87 | 10 ± 3 | 10 | 761 - 1 250 | 1 800 |
| Si | 6 800 ± 8 800 | 4 990 ± 42 | 10 100 | - | - | 6 250 - 6 320 | - |
| Ti | 100 ± 100 | - | 180 | - | - | 135 - 187 | - |
| As | 0.304 ± 0.15 | 0.3 ± 0.1 | - | - | - | 0.52 - 2.25 | - |
| Ba | 450 ± 293 | - | - | - | - | 439 - 669 | - |
| Cd | 8.73 ± 6.73 | 3.6 ± 0.1 | - | 1 210 | 10 | 0.18 - 0.39 | - |
| Co | 10.7 ± 7.78 | 3.2 ± 0.2 | - | - | - | 11.3 - 13.6 | - |
| Cr | 121 ± 79.5 | 76 ± 6 | - | - | 300 | 255 – 5 570 | - |
| Cu | 232 ± 161 | 105 ± 6 | - | 113 ± 3.5 | 110 | 157 - 162 | - |
| Hg | 0.0055 ± 0.0005 | 0.04 ± 0.00 | - | - | - | 0.02 - 0.04 | - |
| Mo | 0.998 ± 0.74 | - | - | - | - | 0.72 - 1.17 | - |
| Ni | 1.19 ± 0.71 | 30 ± 2 | - | - | 200 | 37.7 - 82.6 | - |
| Pb | 19.4 ± 17.9 | 2.4 ± 0.2 | - | - | - | 11.7 - 12.4 | - |
| S | 25 700 ± 14 200 | 8 330 ± 636 | 42 000 | 10 600 ± 500 | 10 600 | 18 300 – 34 500 | - |
| Sr | 412 ± 232 | - | - | - | - | 520 - 639 | - |
| V | 15 ± 4 | - | - | - | - | 2.34 - 3.23 | - |
| Zn | 2 130 ± 1 420 | 733 ± 45 | - | 1 220 ± 21 | 1 200 | 259 - 309 | - |

^a)^ Values presented in Mäkitalo et al 2016 are also presented in Jia et al 2019

^b)^ Data from Sebogodi et al. (2020) is reported in mg/L [suspected to be wrong] but has here been interpreted as mg/kg dw

Table 4 Concentrations of REE and a few other elements in GLD not reported by other references (mg/kg dw)

|  | **This study** | **Golmaei et al. (2018b)** |
| --- | --- | --- |
| Country of origin | Sweden | Finland |
| No of samples | 71 | 1 (n=1) |
| REE | | |
| Er | 0.03 – 0.620 | 0.07 |
| Eu | 0.03 – 0.220 | 0.05 |
| Gd | 0.070 – 0.880 | 0.14 |
| Ho | 0.010 – 0.200 | 0.02 |
| La | 0.600 – 8.900 | 1.6 |
| Lu | 0.010 – 0.090 | <0.01 |
| Nd | 0.100 – 4.700 | 0.9 |
| Pr | 0.100 – 1.450 | 0.3 |
| Sm | 0.080 – 1.030 | 0.14 |
| Tb | 0.010 – 0.150 | 0.02 |
| Tm | 0.010 – 0.070 | <0.01 |
| Y | 0.600 – 6.100 | 0.86 |
| Yb | 0.040 – 0.250 | 0.06 |
| Y | 0.600 – 6.100 | 0.86 |
| Other trace elements | | |
| Ag | 0.72 – 22.05 | 2.2 |
| Th | 0.050 – 0.860 | 0.2 |
| U | 0.050 – 5.040 | 0.2 |
| Rb | 2.2 – 163.2 | 44.1 |
