# Supplementary figures and images for: Chemical characterization of green liquor dregs from 16 Swedish pulp and paper mills between 2017 and 2019
Source: Environ Sci Pollut Res Int. 2024 Jul 3;31(32):45011–34. doi: 10.1007/s11356-024-34074-3 (PMC11254974; doi:10.1007/s11356-024-34074-3)

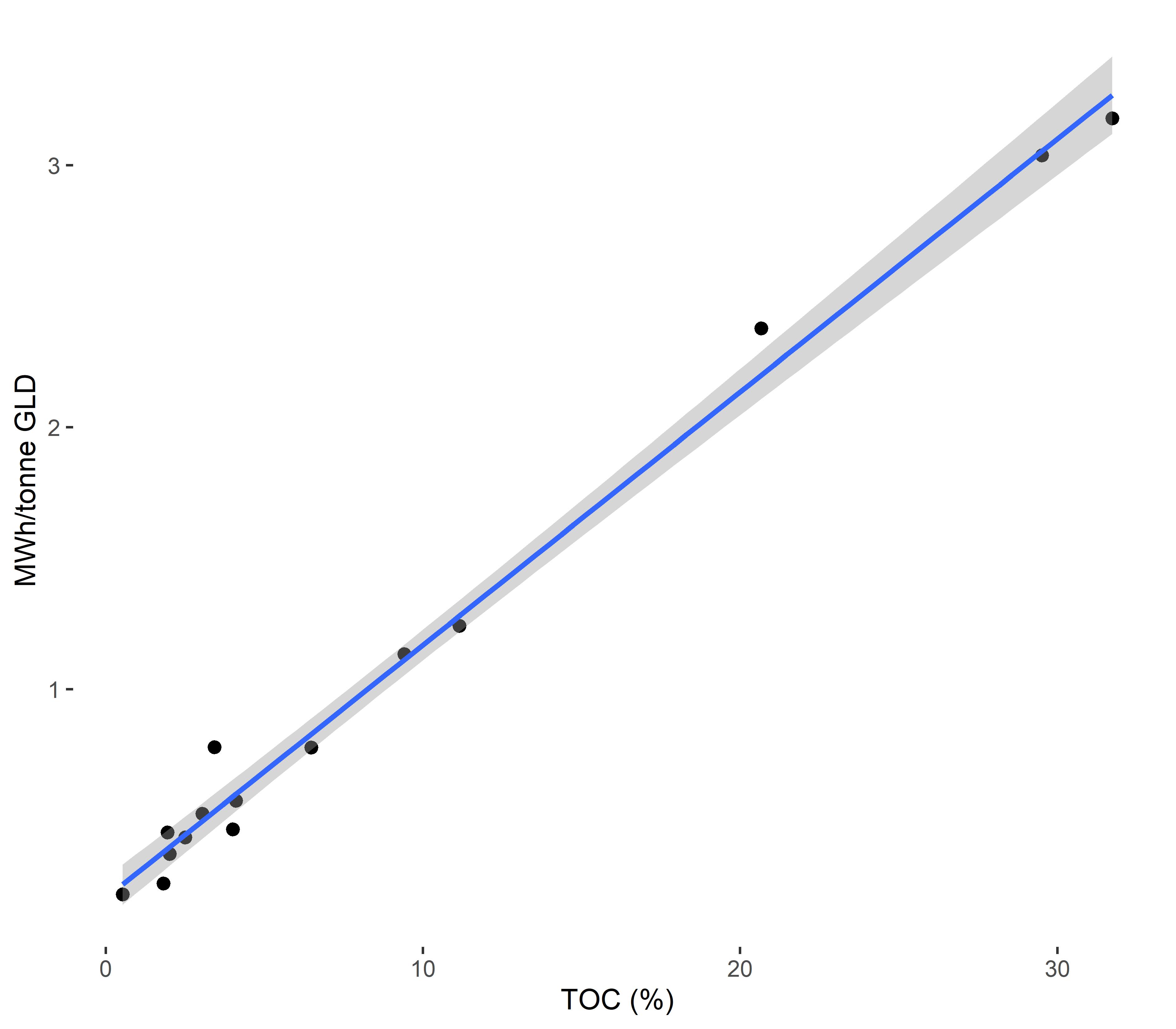

Supplement: Supplementary file 2 — Supplementary file2 (JPEG 272 KB) [file 11356_2024_34074_MOESM2_ESM.jpeg]
